# Phylogeography of sugar kelp: Northern ice‐age refugia in the Gulf of Alaska

**DOI:** 10.1002/ece3.7368

**Published:** 2021-03-19

**Authors:** William Stewart Grant, Erica Chenoweth

**Affiliations:** ^1^ Alaska Department of Fish and Game Anchorage AK USA

**Keywords:** chloroplast DNA, Cordilleran ice sheet, microsatellite DNA, mitochondrial DNA, Northeastern Pacific, phylogeography, Pleistocene ice ages, refugia, *Saccharinalatissima*, sugar kelp

## Abstract

Many Northeast (NE) Pacific fishes and invertebrates survived Pleistocene glaciations in northern refugia, but the extent that kelps survived in northern areas is uncertain. Here, we test the hypothesis that populations of sugar kelp (*Saccharina latissima*) persisted in the Gulf of Alaska during ice‐age maxima when the western margin of the Cordilleran ice sheet covered coastal areas around the NE Pacific Ocean. We estimated genetic diversities within and phylogeographical relationships among 14 populations along 2,800 km in the NE Pacific and Bering Sea with partial sequences of mitochondrial DNA 5′‐cytochrome oxidase subunit I (COI, *bp* = 624, *n* = 543), chloroplast DNA ribulose‐1,5‐bisphosphate carboxylase large subunit‐3′ (*rbc*L, *bp* = 735, *n* = 514), and 11 microsatellite loci. Concatenated sequences of *rbc*L and *COI* showed moderate levels of within‐population genetic diversity (mean *h* = 0.200) but substantial differences among populations (Φ_ST_ = 0.834, *p* < .0001). Microsatellites showed moderate levels of heterozygosity within populations (mean *H*_E_ = 0.391). Kelps in the same organellar lineage tended to cluster together, regardless of geographic origins, as indicated in a principal coordinate analysis (PCoA) of microsatellite genotypes. The PCoA also showed evidence of nuclear hybridizations between co‐occurring organellar lineages. Individual admixture plots with population clusters of *K* = 2, 6, and 9 showed increasing complexity with considerable historical admixture between some clusters. A time‐calibrated phylogeny placed divergences between *rbc*L‐*COI* lineages at 1.4 million years at most. The time frames of mutation in the *rbc*L‐*COI* lineages and microsatellite population clusters differed among locations. The existence of ancient lineages in the Gulf of Alaska, moderate levels of genetic diversity, and the absence of departures from neutrality are consistent with northern refugia during multiple Croll‐Milankovitch climate cycles in the Pleistocene Epoch.

## Graphical abstract

Organellar *COI* and *rbc*L sequences and 11 microsatellite loci were assayed in over 500 plants from 14 localities to test the hypothesis that populations survived Pleistocene ice ages in northern refugia. The substantial genetic diversity and chaotic distribution of genetic markers among populations indicated the likely survival in multiple northern refugia.

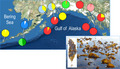

## 1 INTRODUCTION

A continuing challenge to evolutionary biologists is to identify environmental variables that shape the genetic structures of natural populations through the actions of gene flow, random genetic drift, and natural selection, all of which operate against a backdrop of deep genetic structure created by historical events (Marko & Hart, 2011). A major source of deep structure in temperate and boreal species has been isolations in glacial refugia and vicariant separations forced by massive sheets of ice that spread over North America and Eurasia during the Pleistocene Epoch (Raymo, 1994; Stewart et al., 2010). A widely held view, based on phylogeographic patterns in terrestrial species, posits that northern populations contracted into southern ice‐free refugia as glaciers advanced and then expanded northwards as glaciers receded (Hewitt, 1996, 2000). A consequence of these contractions and expansions was the loss of genetic diversity in northern populations as a result of serial, postglacial colonizations (Hewitt, 1996). In contrast, some terrestrial species appear to have survived in northern refugia without losing genetic diversity (Birks et al., 2005; Stewart & Lister, 2001). Populations surviving in northern refugia may also show phylogeographic breaks with southern populations, as well as heterogeneity among populations resulting from isolations in local refugia.

The shores of the Northeastern (NE) Pacific were periodically covered by the margins of the Cordilleran ice sheet in the Pleistocene Epoch from 2.6 to 0.012 million years (Ma) during cold depressions in 40–100 thousand year climate cycles (Li & Born, 2019; Rasmussen et al., 2014). Since ice margins were irregular, a patchwork of coastal habitats between lobes of glacial ice potentially served as refugia (Carrara et al., 2007; Kaufman & Manley, 2004). Concurrently, global sea levels during glacial maxima dropped as much as 120 m below present‐day levels (Rabineau et al., 2006; Rohling et al., 1998), re‐sculpting shorelines. Unlike terrestrial refugia, the sizes, number, and locations of coastal refugia, and postglacial dispersal pathways were limited by the more or less linear nature of shorelines. Survival in northern marine glacial refugia has been suggested for several intertidal and shallow‐water species of algae (Lindstrom et al., 1997) and a variety of invertebrates (Marko et al., 2010; Marko & Zaslavskaya, 2019). While numerous genetic studies have been made of kelps along the coasts of Washington, Oregon, and California (*e.g*., Alberto et al., 2010, 2011), few population studies have been made at higher latitudes.

**FIGURE 1:**
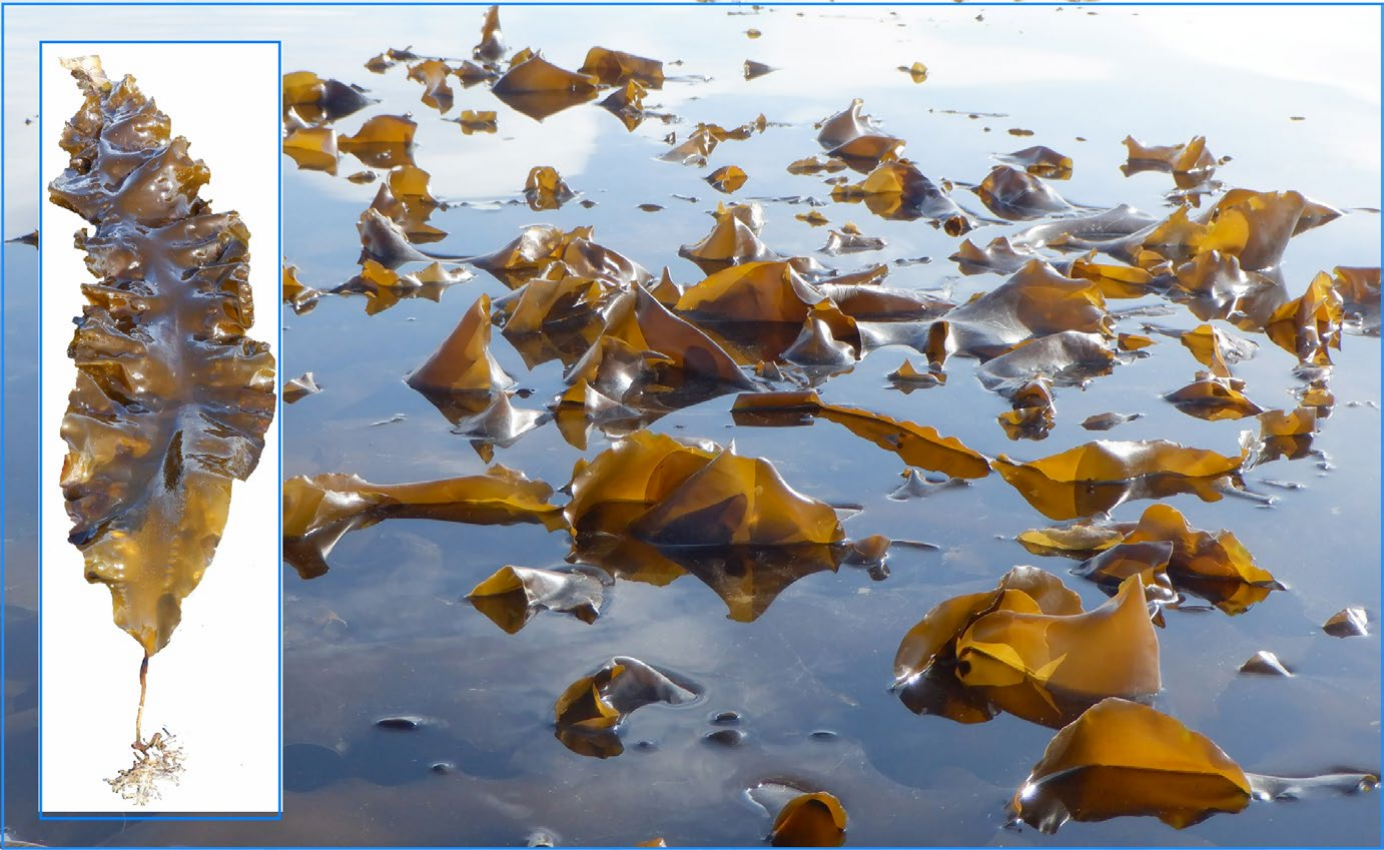
Photographs of sugar kelp *Saccharina latissima*. Left: Recruit of the year about 1.5 m in length. Right: Blades of sugar kelp at low tide in a wave‐protect area behind the breakwater at Homer Spit, Alaska

Here, we focus on populations of sugar kelp (*Saccharina latissima* Lane, Mayes, Druehl & Saunders) (Figure 1), a species distributed from Central California through the Arctic and into the North Atlantic (Bringloe et al., 2017; Neiva et al., 2018). Several biological features of sugar kelp bear on understanding its phylogeographic structure in the Gulf of Alaska. Sugar kelp occupy a narrow ecological niche, inhabiting low intertidal and shallow subtidal areas in wave‐protected coves or bays and growing well only at 5–17°C in temperate regions (Druehl, 1967; Machalek et al., 1996). Nevertheless, some varieties of this kelp tolerate colder temperatures in Arctic waters (Bringloe et al., 2017; Neiva et al., 2018). Abundance and reproductive output vary greatly among sites and years, so that populations can be ephemeral on decadal time scales (Bekkby & Moy, 2011; A. Raymond, pers. comm.). Individual kelps tend to be perennial in the North Atlantic, but largely annual in Alaska (Bartsch et al., 2008; A. Raymond, pers. comm.).

The reproductive biology of sugar kelp influences connectivity between populations. This kelp alternates between large‐bladed sporophytic kelps (Figure 1), anchored to small rocks and pebbles with branched haptera, and a microscopic, filamentous gametophyte phase (Lindeberg & Lindstrom, 2010). Sporophytes (2*n* chromosome complement) produce large numbers of meiospores, which settle after a brief planktonic phase and sprout into haploid, filamentous gametophytes with an *n* chromosomal complement. Male gametophytes produce spermatozoa that fertilize oogonia on female gametophytes, producing zygotes that grow in place into large‐bladed sporophytic kelps. Even though spores can potentially be transported in coastal currents for several days (van den Hoek, 1987), realized spore dispersals of kelps are generally limited to only a few meters (Anderson & North, 1966; Dayton, 1985; Santelices, 1990; Stein et al., 1995). Nevertheless, the drifting of reproductively mature sporophytes along a shore may occasionally contribute to long‐distance dispersals (Saunders, 2014).

The goal of this study was to search for genetic imprints in contemporary populations that would shed light on historical events in the Gulf of Alaska in the Pleistocene Epoch. Partial sequences of genes encoded in mitochondrial (mt) and chloroplast (cp) DNAs were used to reconstruct gene genealogies and test for genetic population structure. We also used microsatellite DNA markers to estimate recent processes influencing genetic variability (Paulino et al., 2016). The expected smaller mutation rates of the two organellar genes potentially resolve deep population events, whereas the apparent larger mutation rates at microsatellite loci potentially resolve contemporary population dynamics. These data together were used to test whether sugar kelp populations survived in southern refugia, or in local refugia in the Gulf of Alaska.

## 2 MATERIALS AND METHODS

### 2.1 Kelp collections and DNA extraction

Several authors have noted misidentifications between split kelp *Hedophyllum nigripes* (as *Laminaria groenlandica*, *Saccharina groenlandica*, *Saccharina nigripes*) and sugar kelp *Saccharina latissima* (*Laminaria latissima*, *Laminaria saccharina*) (Bartsch et al., 2008; Grant et al., 2020; Longtin & Saunders, 2015). In our study, misidentifications of *S. latissima* were discovered through molecular analysis of mtDNA 5′‐cytochrome oxidase (*COI*). Young individuals of *H. nigripes* and *S. latissima* generally have bullated blades and similar morphologies. However, the blades of older *H. nigripes* are leathery and slippery to the touch, whereas those of *S. latissima* are thinner and lack mucilaginous glands (Longtin & Saunders, 2016). In the Gulf of Alaska, *H. nigripes* occurs on only wave‐exposed or current‐swept rocky shores consisting of bedrock or large boulders, whereas *S. latissima* occurs largely in wave‐protected inlets and coves and is usually attached to small rocks and pebbles on a sedimentary bottom. While the two kelps can occur along the same stretch of beach, they are segregated into exposed (*H. nigripes*) and protected (*S. latissima*) microhabitats. A similar association between wave exposure and the occurrences of *H. nigripes* (as *Saccharina nigripes*) and *S. latissima* was found in the Bay of Fundy, in the NW Atlantic Ocean (Longtin & Saunders, 2016).

A 4‐cm^2^ piece of frond near the basal meristem was excised from sporophytes, damp‐dried, and immediately desiccated with silica beads. Kelps at least 1 m apart were collected to avoid sampling siblings, or closely related individuals. DNA was extracted from 10 to 20 mg of dried tissue with a NucleoSpin^®^ 96 Plant II Kit (Macherey‐Nagel Inc., Düren, Germany). Standard extraction kit protocols were followed, except dried tissues were homogenized at room temperature by crushing or chopping on weighing paper with a scalpel.

### 2.2 Nucleotide sequencing of organellar DNAs

A segment of mtDNA *COI*‐5′ was amplified by PCR with forward *GazF2* (5′CCAACCAYAAAGATATWGGTAC3′) (Bittner et al., 2008) and reverse *GazR2* (5′GGATGACCAAARAACCAAAA3′) (Burrowes et al., 2003) primers from Lane et al. (2007). A segment of cpDNA ribulose‐1,5‐bisphosphate carboxylase/oxygenase large subunit‐3′ (*rbc*L) was amplified with the forward *rbc*L‐*543F* (5′CCWAAATTAGGTCTTTCWGGWAAAAA3′) and reverse *rbc*L‐*1381R* (5′ATATCTTTCCATARRTCTAAWGC3′) primers (Silberfeld et al., 2010). Extracts were diluted 100‐fold with deionized water before amplification to avoid problems with polysaccharides exuded from fronds. PCR cocktails consisted of a 50 μl mixture of 2.0 μl template DNA in 1× Colorless GoTaq Flexi buffer, 2.5 mM MgCl_2_, 0.2 mM of each dNTP, 1 μM each of forward and reverse primers, and 2.5 U GoTaq Flexi DNA polymerase. GeneAmp PCR System 9700 thermocyclers (ABI 9700; Applied Biosystems, Inc., Foster City, CA) were used to amplify DNA with an initial denaturation at 94°C for 3 min and 35 cycles of 94°C for 45 s, 50°C (*COI*), or 52°C (*rbc*L), for 1 min (primer annealing), 72°C for 1.5 min, and a final step at 72°C for 5 min.

Amplified DNAs were routinely sequenced in forward and reverse directions by GENEWIZ Inc. (South Plainfield, NJ), or by the University of Arizona Genetics Core (Flagstaff, AZ). Forward and reverse complement sequences were aligned and edited with MEGA 7.0.20 (Kumar et al., 2016), and chromatograms were viewed with Finch TV 1.4.0 (Geospiza Inc.). After editing, 624 bp of *COI* and 735 bp of *rbcL* were used for population analyses. Quality control consisted of re‐extracting and re‐sequencing unique haplotypes from each 96‐well plate.

### 2.3 Microsatellite genotyping

We used 11 microsatellite loci, *SLN32*, *SLN34*, *SLN36*, *SLN54*; *SLN58*, *SLN62, SLN314*, *SLN319*, *SLN320*, *SLN510,* and *SLN511*, previously developed for North Atlantic *S. latissima* (Paulino et al., 2016). PCR was used to amplify microsatellite alleles with ABI 9700 thermocyclers (Applied Biosystems, Inc., Foster City, CA). Each 10 µl PCR cocktail consisted of 2 µl template DNA diluted fourfold in deionized water mixed with (~0.1 µg/µl) 1× Colorless GoTaq Flexi Buffer (Promega Inc., Madison, WI), 1.5–2.5 mM MgCl_2_ (Promega Inc., Madison, WI), 0.20 mM of each nucleotide (Applied Biosystems, Inc.), 0.10–0.25 µM of forward and reverse primers, 0.1 mg/ml of BSA (Sigma Inc., St. Louis, MO), 0.05 U GoTaq Flexi DNA polymerase (Promega Inc., Madison, WI), and deionized water. Thermal cycling profiles varied with locus (Table S1 in Appendix S1), and appropriate dyes were used to tag the amplified DNA fragments (Table S2 in Appendix S1). Amplicons were electrophoretically separated by size in an Applied Biosystems 3730 capillary DNA sequencer. Genotypes were scored with GeneMapper 5.0 (Applied Biosystems) independently by two technicians. 8% of the samples was re‐extracted and re‐genotyped by a third technician for quality control.

### 2.4 Statistical analyses

#### 2.4.1 Organellar DNA

We used arlequin 3.5.2.2 (Excoffier & Lischer, 2010) to estimate the number of polymorphic nucleotide sites, *N*_poly_, observed, *N*_H_, and expected, *N*_EH_, haplotypes under neutrality, gene diversity, *h* (standard deviation), nucleotide diversity, *θ*_π_ (standard deviation), and number of private haplotypes in a sample, *N*_PH,_ for *COI* and *rbc*L sequences. Departures from neutrality were tested with Tajima's *D* (Tajima, 1989). Sequence divergences between populations were estimated with *F*_ST_ (Weir & Cockerham, 1984) and Φ_ST_ with appropriate mutation models as determined with MEGA 7 (Kumar et al., 2016). IBD 1.52 (Bohonak, 2002) was used to test for isolation by distance with Mantel's test between difference matrices of pairwise genetic distances, corrected for diversity [Φ_ST_/(1 − Φ_ST_)] for organellar DNA or [*F*_ST_/(1 − *F*_ST_)] for microsatellites, and approximate shoreline distances between samples. Tests were made with and without log transformations of geographic distances and with 1,000 randomizations.

A Bayesian tree of evolutionary relationships was produced from unique *rbc*L‐*COI* haplotypes with BEAST 1.8.4 (Drummond & Rambaut, 2007), with a strict clock, the Yule model of speciation, and the HKY+G mutation model. Nodes in the tree were time‐calibrated with a divergence of 6.9 Ma between *S. latissima* and *S. japonica*, as indicated in Figure 2 of Starko et al. (2019), with a normal distribution and standard deviation of 0.7. Ten million MCMC steps produced effective sample sizes (ESS) of well over 200 for each tree‐related component.

We also considered using such programs as BEAST to produce the Bayesian skyline plots (Drummond et al., 2005), or Ima2 (Hey & Nielsen, 2004), to reconstruct demographic history. However, the data at hand for sugar kelp are not appropriate for these analyses, as sample sizes for discrete populations of randomly mating individuals are too small. Pooling of genetically heterogeneous populations to achieve larger sample sizes would yield misleading results (Grant, 2015). Additionally, kelps show reproductive skew and hence do not follow the Wright–Fisher model of coalescence used in these programs for simulations to estimate medians and credibility intervals (Eldon & Wakeley, 2006; Grant et al., 2016). Beyond these technicalities, the effects of population growth cannot clearly be distinguished from the effects of reproductive skew on the nucleotide site frequency spectrum, which forms the basis of these analyses (Niwa et al., 2016).

#### 2.4.2 Microsatellite DNA

We used GENEPOP 4.6 (Rousset, 2008) to test for fit to Hardy–Weinberg genotypic proportions, using Markov chain Monte Carlo chains of 10,000 steps in 100 batches and a Bonferroni correction (Rice, 1989) of *p* = .05/11 = .0045 to control type I error at *α* = 0.05. We used gene diversity analysis (Lewis & Zaykin, 2002) to estimate observed (*H*_O_) and expected (*H*_E_) heterozygosity averaged over loci, to count the number of alleles at each locus, and to estimate the inbreeding coefficient, *F*_IS_. HP‐RARE (Kalinowski, 2005) was used to estimate allelic richness based on the smallest sample size. We used ML‐Null (Kalinowski & Taper, 2006) and GENEPOP to estimate null‐allele frequencies.

We examined the geographic and genetic components of population structure with four approaches. GENALEX 6.503 (Peakall & Smouse, 2012) was used to define principal coordinate analysis (PCoA) of allele frequency variability among samples with standardized covariance and with option to estimate missing genotypes. GENALEX was also used to reassign individuals to populations with significance of likelihoods set to 0.01 and to compute an AMOVA of allelic frequencies to estimate the overall levels of diversity within and among populations with 50,000 permutations.

STRUCTURE (Pritchard et al., 2000) was used to estimate population clusters and admixtures of individuals from hybridizations between clusters. We searched for the best fit of the data with population groups ranging from *K* = 1 to 10 with 10 replicates of 5,000 burn‐in and 50,000 MCMC steps for each value of *K*. We used a uniform prior of admixture, assumed a correlation of allele frequencies among populations, and estimated the probability (maximum likelihood) of the data under the model. Web‐based STRUCTURE HARVESTER (Earl & vonHoldt, 2012) was used to summarize the likelihoods of *K* in the various runs with the approach of Evanno et al. (2005) and to produce estimates of Δ*K*. Meirmans (2015) has cautioned that since the *K* statistics are “dubious at best” and “ad hoc,” more than one value of *K* may be of evolutionary relevance.

The model used in STRUCTURE assumes that (1) loci are unlinked, (2) loci are at linkage equilibrium within population clusters, and (3) genotypes in population clusters are in Hardy–Weinberg proportions. To begin, we tested for linkage between loci overall and in the sampled populations with GENEPOP. We found that 2 locus pairs were significantly linked over all (Table S3 in Appendix S1) and 17 of 715 pairs tested showed significant disequilibrium before applying a correction for false positives (Table S4 in Appendix S1). Hence, there was no indication of physical linkage between pairs of loci: The locus pairs showing disequilibria within populations were scattered among populations and were likely due to demographic history, or to chance. The inbreeding coefficient, *F*_IS_, ranged from −0.052 to 0.096, except for one population for which it was 0.217, and averaged 0.038. Hence, the populations did not substantially depart from the Hardy–Weinberg expectations.

## 3 RESULTS

We present the results for each of the organellar markers separately to facilitate comparisons with other studies, but use the results for concatenated *rbc*L‐*COI* sequences to make our major inferences about population structure.

### 3.1 Cytochrome oxidase‐5′ (*COI*)

**FIGURE 2:**
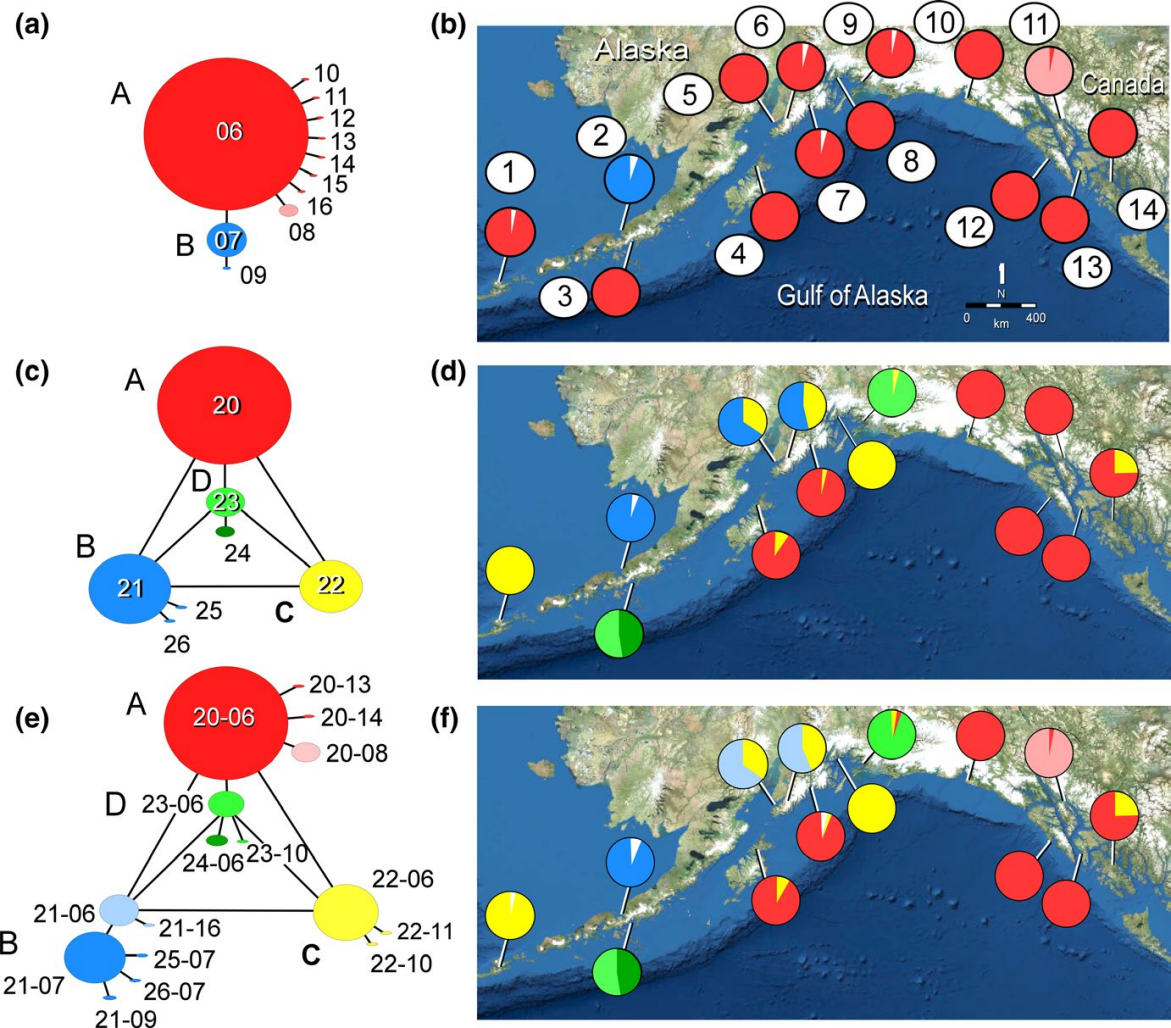
Haplotype networks of organellar variability and maps depicting haplotype frequencies among populations of sugar kelp, *Saccharina latissima*, in Alaska. (a, b) *COI* (624 bp), haplotype numbers correspond to the last two digits of GenBank Accession Nos. MT040306–MT040316; (c, d) *rbc*L (735 bp) haplotype numbers correspond to the last two digits of GenBank Accession Nos. MT040320–MT040326; and (e, f) concatenated *COI‐rbc*L (1,359 bp), haplotype designations as in Table 2. White wedges represent private haplotypes found at a single site. Location numbers as in Table 1

Sample sizes ranged from 6 to 90 and averaged 33 kelps (Tables S5 and S6 in Appendix S1). Ten nucleotide polymorphisms in a 624 bp fragment of *COI* defined 11 haplotypes, but only three haplotypes were abundant. A central abundant haplotype (MT040306 in 73.1% of kelps) was connected to 9 peripheral haplotypes by one mutation (Figure 2a). This lineage corresponds to lineage “A” of Neiva et al. (2018). All or nearly all of the kelps in 12 of the 14 samples carried the same common haplotype (“fixed”) (Figure 2b). However, kelps from Port Moller (sample 2) carried a unique haplotype (MT040307) that was one mutation removed from the central haplotype, and kelps from Auke Bay (11) carried another haplotype (MT040308) also one mutation removed from the central haplotype (Figure 2a,b). Six of the samples had one or two private haplotypes that were one mutation removed from the central haplotype.

Haplotype diversity (*h*) ranged from 0.0 in 8 samples to 0.145 in sample 7 and was 0.429 ± 0.024 overall (Table S6 in Appendix S1). Nucleotide diversity (θ_π_) ranged from 0.0 in several samples to 0.029% in sample 9 and was 0.079% ± 0.076% in the pooled sample. Tajima's *D* was marginally significant at three locations and in the pooled sample (*D* = −1.452, *p* = 0.041). Overall, the number of observed haplotypes (*N*_H_) was 11, when only 4.39 were expected under neutrality (*N*_EH_). A total of 10 private haplotypes appeared at six locations.

Genetic divergence (Φ_ST_) between populations ranged from 0.0 between pairs fixed with the same haplotype to 0.968 between populations fixed, or nearly fixed, with different haplotypes (Table S7 in Appendix S1). 90.9% of the overall diversity was due to differences among populations, and 9.1% was contained within populations as different haplotypes among plants (Table S8 in Appendix S1).

### 3.2 Ribulose‐1,5‐bisphosphate carboxylase/oxygenase large subunit (*rbc*L)

Sample sizes ranged from 6 to 81 and averaged 32.1 kelps (Table S9 in Appendix S1). Four polymorphic nucleotide sites in a 735 bp fragment of *rbc*L defined 7 haplotypes in 4 lineages, **A**, **B**, **C**, and **D** (Figure 2c). The four most abundant haplotypes were separated from one another by substitutions at a single nucleotide site that was in the third position of a codon. Mutations between six pairs of haplotypes involved both transitions (MT040320‐MT040322, C ↔ T; MT040321‐MT040323, G ↔ A) and transversions (MT040320‐MT040321, C ↔ G; MT0040320‐MT040323, C ↔ A; MT040321‐MT040322, G ↔ T; MT040322‐MT040323, T ↔ A). The most abundant haplotype (MT040320) appeared at 7 locations, was fixed at 4 locations and was at a high frequency at 3 locations (Figure 2d). The distribution of this haplotype was geographically disjunctive, appearing in the central Gulf of Alaska (4 & 7) and in Southeast Alaska (10–14). The second most abundant haplotype (MT040321) appeared at 3 locations, Port Moller (2) and Kachemak Bay (5 and 6). Haplotype MT040322 appeared in the eastern Aleutians (1), Kachemak Bay (5 and 6), and Prince William Sound (8). Haplotype MT040323 appeared disjunctively at Sand Point (3) but also several hundred km away at Cordova (9).

The overall number of *rbc*L haplotypes was 7, but the expected number under neutrality was 10.5. Haplotype diversity (*h*) ranged from 0.0 in 6 samples to 0.515 ± 0.027 in sample 3 and was 0.702 ± 0.013 in the pooled sample (Table S10 in Appendix S1). Nucleotide diversity (θ_π_) ranged from 0.0 in 6 samples to 0.070% ± 0.067% and was 0.104% ± 0.085 in the pooled sample. Tajima's *D* was not significant in any of the samples, nor in the pooled sample (*D* = 0.453, *p* = 0.716).

Divergences (Φ_ST_) between populations ranged from 0.0 between populations with the same haplotype to 1.0 between populations with different haplotypes (Table S11 in Appendix S1). Overall, Φ_ST_ = 0.788 (*p* < 0.00005) among samples. AMOVA indicated that 78.8% of the total variation was due to differences among populations and 21.2% was due to differences among kelps within populations (Table S12 in Appendix S1).

### 3.3 Concatenated *COI* and *rbc*L

Sample sizes of the concatenated sequences were slightly smaller than those of the two genes individually, because both genes were not successfully sequenced in some kelps. A total of 13 polymorphic sites defined 16 haplotypes in 447 sequences from 14 locations (Figure 2e, Tables 1 and 2). The number of haplotypes averaged 2.2 per sample, and the expected number of haplotypes under neutrality was 1.87. *h* ranged from 0.0 in 4 populations to 0.574 in population 6 and averaged 0.200. Nucleotide diversity (θ_π_) ranged from 0.0% to 0.048% and averaged 0.016%. Tajima's *D* was significant (*p* < 0.05) in populations 2, 7, and 9, but was not significant overall (*p* = 0.203).

**TABLE 1:** Sample information and estimates of genetic parameters based on concatenated fragments of mitochondrial DNA cytochrome oxidase I‐5′ (*COI*) and ribulose‐1,5‐bisphosphate carboxylase/oxygenase large subunit‐3′ (*rbc*L) (1,359 base pairs combined) in samples from the Gulf of Alaska and southeastern Bering Sea (locations 1–14)

Location	N lat.	Long.	*N*	*N*_poly_	*N*_H_	*N*_EH_	*N*_PH_	*h*	θ_π_ (%)	*D*	*p*
1 Nateen Bay, Unalaska	53.883	−166.634	37	3	2	1.18	1	0.054 ± 0.051	0.012 ± 0.019	−1.722	.012
2 Port Moller, Alaska Peninsula	55.989	−135.278	80	5	4	1.49	4	0.121 ± 0.049	0.026 ± 0.029	−1.423	.046
3 Kuiuk Bay, Alaska Peninsula	56.179	−158.520	30	1	2	3.48	1	0.515 ± 0.027	0.038 ± 0.036	1.621	.976
4 Malina Bay, Kodiak Island	58.176	−152.995	28	0	1	1.00	0	0.0	0.0	–	–
5 Homer Spit, Kachemak Bay	59.604	−151.418	30	1	2	3.06	0	0.460 ± 0.061	0.034 ± 0.034	1.280	.919
6 Humpy Creek, Kachemak Bay	59.668	−151.135	31	3	4	4.06	2	0.574 ± 0.048	0.047 ± 0.042	−0.342	.402
7 Lowell Point, Resurrection Bay	60.032	−149.437	27	3	4	1.72	2	0.214 ± 0.103	0.016 ± 0.022	−1.734	.014
8 Whittier, Prince William Sound	60.787	−148.634	6	0	1	1.00	0	0.0	0.0	–	–
9 Cordova, Prince William Sound	60.545	−145.768	22	2	3	1.55	1	0.178 ± 0.106	0.013 ± 0.020	−1.515	.041
10 Boat Harbor, Yakutat	59,563	−139.743	29	0	1	1.00	0	0.0	0.0	–	–
11 Auke Bay, Juneau	58.376	−134.702	26	1	1	1.23	1	0.077 ± 0.070	0.006 ± 0.001	−1.156	.139
12 Harris Island, Sitka	57.036	−135.278	23	0	1	1.00	0	0.0	0.0	–	–
13 Tokeen Bay, Scott Island	55.893	−133.383	31	0	1	1.00	0	0.0	0.0	–	–
14 Kaguk Bay, Prince of Wales Island	55.745	−133.288	46	1	2	3.12	0	0.433 ± 0.055	0.032 ± 0.032	1.239	.908
Mean	–	–	31.9	1.4	2.1	1.85	0.9	0.188	0.016	–	–
Pooled	–	–	446	13	16	14.77	12	0.781 ± 0.012	0.088 ± 0.063	−0.868	.208

Note

Location number, sample size (*N*), number of polymorphic nucleotide sites (*N*_poly_), number of haplotypes (*N*_H_), expected number of haplotypes under neutrality (*N*_EH_), number of private haplotypes (*N*_PH_), haplotype diversity (*h* ± standard deviation), nucleotide diversity (θ_π_ ± standard deviation), and Tajima's *D* (*p*: probability of null hypothesis of neutrality).

**TABLE 2:** Haplotype frequencies of concatenated fragments of ribulose‐1,5‐bisphosphate carboxylase/oxygenase large subunit‐3′ (*rbc*L) and mitochondrial DNA cytochrome oxidase I‐5′ (*COI*) (1,359 bp) in samples from the Gulf of Alaska and southeastern Bering Sea (samples 1–14)

Haplotype	Lineage	Location														Total
		1	2	3	4	5	6	7	8	9	10	11	12	13	14	
20‐06	A	.	.	.	28	.	.	24	.	.	29	1	23	31	32	168
20‐07	B	.	75	.	.	.	.	.	.	.	.	.	.	.	.	75
22‐06	C	36	.	.	4	10	13	1	6	1	.	.	.	.	10	81
23‐06	D	.	.	16	.	.	.	.	.	20	.	.	.	.	.	36
21‐06	B	.	.	.	.	20	16	.	.	.	.	.	.	.	.	36
20‐08	A	.	.	.	.	.	.	.	.	.	.	25	.	.	.	25
27‐06	D	.	.	14	.	.	.	.	.	.	.	.	.	.	.	14
21‐09	B	.	3	.	.	.	.	.	.	.	.	.	.	.	.	3
25‐07	B	.	2	.	.	.	.	.	.	.	.	.	.	.	.	2
26‐07	B	.	1	.	.	.	.	.	.	.	.	.	.	.	.	1
22‐10	C	1	.	.	.	.	.	.	.	.	.	.	.	.	.	1
22‐11	A	.	.	.	.	.	1	.	.	.	.	.	.	.	.	1
21‐12	A	.	.	.	.	.	1	.	.	.	.	.	.	.	.	1
20‐13	A	.	.	.	.	.	.	1	.	.	.	.	.	.	.	1
20‐14	A	.	.	.	.	.	.	1	.	.	.	.	.	.	.	1
23‐15	A	.	.	.	.	.	.	.	.	1	.	.	.	.	.	1
Total		37	81	30	32	30	31	27	6	22	29	26	23	31	42	447

Note

Haplotype designations consist of the last two digits of the GenBank Accession Numbers for *rbc*L‐*COI* and correspond to haplotype designations in Figure 2e. Sample numbers as in Table 1.

**FIGURE 3:**
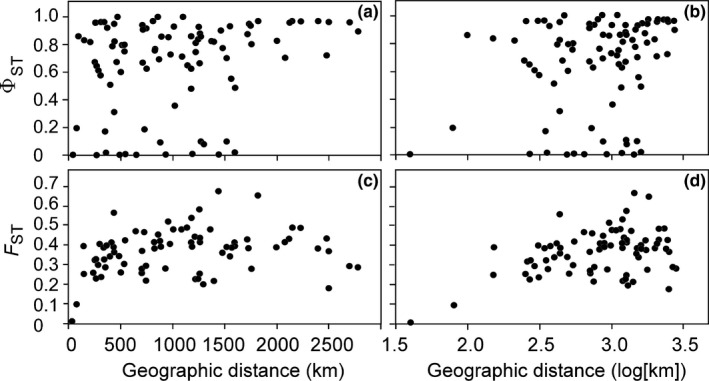
Mantel's tests for isolation by distance between populations in the Gulf of Alaska and southeastern Bering Sea. Concatenated *COI*‐*rbc*L sequences (1,359 bp): (a) Φ_ST_/(1 − Φ_ST_) and geographical distance (km) *r* = 0.264, *p* = 0.027, and (b) Φ_ST_/(1 − Φ_ST_) and log10 (km) *r* = 0.246, *p* = 0.027. Microsatellite DNA: (c) *F*_ST_/(1 − *F*_ST_) and geographical distance *r* = 0.231, *p* = 0.080, and (d) *F*_ST_/(1 − *F*_ST_) and log10 (km) *r* = 0.401, *p* = 0.005

Φ_ST_ ranged from 0.0 between populations fixed with the same haplotype to 0.971 between populations 1 and 13 (Table 3). A majority of population pairs (66 of 78) showed significant sequence divergences. AMOVA indicated that 83.4% of the variability was due to differences among populations on average, and 16.6% was contained within populations (Table S13 in Appendix S1). A weak, but significant signal of isolation by distance appeared among populations with geographic shoreline distances in km (Mantel's *r* = 0.264, *p* = 0.027; Figure 3a) and in log(km) (*r* = 0.246, *p* = 0.027; Figure 3b).

**TABLE 3:** Lower triangle: genetic distances (Φ_ST_) between samples listed in Table 1 based on concatenated fragments of mitochondrial DNA 5′‐cytochrome oxidase (*COI*) and ribulose‐1,5‐bisphosphate carboxylase/oxygenase large subunit‐3′ (*rbc*L) (1,359 base pairs) with the Tamura and Nei (1993) model of mutation

1	–	0.362	0.564	0.519	0.489	0.536	0.581	0.675	0.654	0.490	0.489	0.383	0.432	0.362
2	**0.952**	–	**0.274**	**0.241**	**0.227**	**0.217**	**0.342**	**0.380**	**0.432**	**0.367**	**0.179**	**0.290**	**0.286**	**0.274**
3	**0.823**	**0.907**	–	**0.301**	**0.261**	**0.293**	**0.383**	**0.404**	**0.479**	**0.426**	**0.277**	**0.389**	**0.415**	**0.301**
4	**0.851**	**0.926**	**0.750**	–	**0.229**	**0.238**	**0.282**	**0.261**	**0.419**	**0.419**	**0.198**	**0.393**	**0.407**	**0.229**
5	**0.651**	**0.838**	**0.668**	**0.645**	–	**0.009**	**0.258**	**0.342**	**0.415**	**0.410**	**0.227**	**0.360**	**0.383**	*0.009*
6	**0.478**	**0.819**	**0.622**	**0.572**	0.0	–	**0.297**	**0.388**	**0.452**	**0.441**	**0.253**	**0.388**	**0.398**	**0.297**
7	**0.882**	**0.930**	**0.757**	0.012	**0.675**	**0.607**	–	**0.252**	**0.422**	**0.393**	**0.281**	**0.390**	**0.432**	**0.252**
8	0.0	**0.945**	**0.728**	**0.793**	*0.508*	*0.311*	**0.830**	–	**0.400**	**0.370**	**0.218**	**0.480**	**0.481**	**0.400**
9	**0.900**	**0.935**	**0.356**	**0.801**	**0.674**	**0.599**	**0.818**	**0.859**	–	**0.387**	**0.345**	**0.470**	**0.464**	**0.387**
10	**0.970**	**0.957**	**0.822**	0.099	**0.767**	**0.692**	0.003	**1.000**	**0.925**	–	**0.322**	**0.405**	**0.415**	**0.322**
11	**0.968**	**0.963**	**0.872**	**0.854**	**0.857**	**0.813**	**0.859**	**0.968**	**0.938**	**0.962**	–	**0.322**	**0.405**	**0.415**
12	**0.968**	**0.954**	**0.805**	0.075	**0.746**	**0.667**	0.0	**1.000**	**0.915**	0.0	**0.958**	–	**0.326**	**0.326**
13	**0.971**	**0.957**	**0.827**	0.095	**0.773**	**0.699**	0.005	**1.000**	**0.927**	0.0	**0.964**	0.0	–	0.098
14	**0.721**	**0.897**	**0.704**	0.014	**0.553**	**0.482**	*0.097*	*0.624*	**0.711**	*0.185*	**0.789**	*0.167*	*0.191*	–
	1	2	3	4	5	6	7	8	9	10	11	12	13	14
	Location													

Note

Overall, Φ_ST_ = 0.834 (*p* < 0.001) among populations in the Gulf of Alaska and Southeast Bering Sea. Upper triangle: genetic distances (*F*_ST_) between samples based on 11 microsatellite loci. Overall, *F*_ST_ = 0.239 (*p* < 0.001). Sample numbers as in Table 1. Italics 0.05 > *p* > .01; bold *p* < 0.01. Significances determined with 50,000 permutations.

**FIGURE 4:**
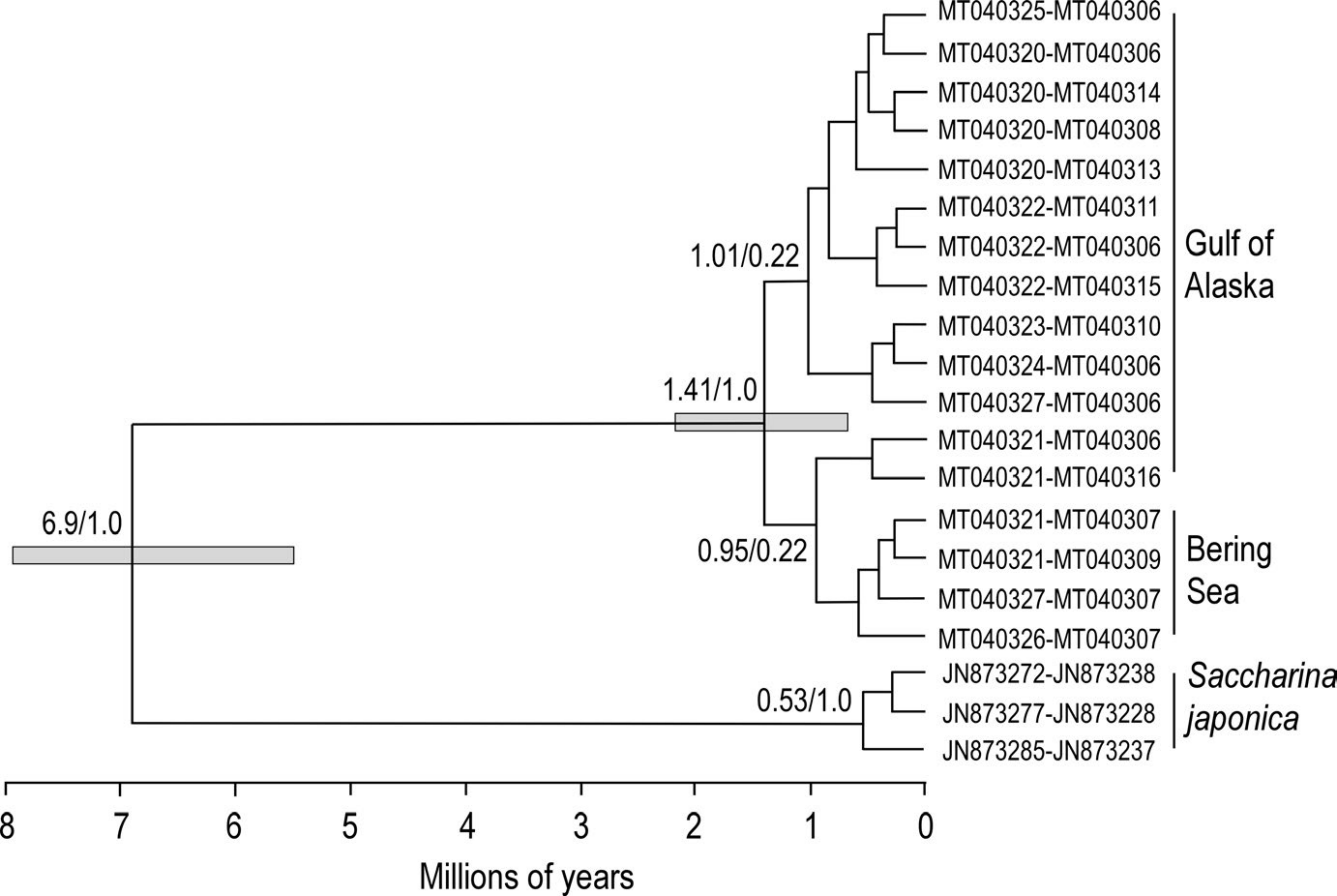
Bayes tree of concatenated *rbc*L and *COI* haplotypes (1,359 bp) of *Saccharina latissima* in the Gulf of Alaska, Bering Sea, and Russia. Numbers at nodes represent estimated age of the node in millions of years (Ma) and the posterior probability of support for the node based on 10 million MCMC trees. Bars represent 95% highest probability densities for the age of a node. The NW Pacific kelp *Saccharina japonica* serves as an outgroup taxon to date the nodes in the tree using a divergence of 6.9 Ma (Starko et al., 2019)

A Bayesian tree of unique *rbc*L‐*COI* haplotypes, rooted by Japanese kelp, *Saccharina japonica*, with a time since divergence of about 6.9 Ma (Starko et al., 2019), indicated that deep divergences between lineages ranged from 0.95 to 1.41 Ma (Figure 4).

### 3.4 Microsatellites

Estimates of null‐allele frequencies with GENEPOP and ML‐null differed considerably, as ML‐null estimated null‐allele frequencies for invariant loci (Tables S14 and S15 in Appendix S1). Estimates of null‐allele frequencies with GENEPOP were systematically smaller than those with ML‐null. For the GENEPOP results, no locus nor population stood out with consistently large frequencies of null alleles. Hence, we used the entire microsatellite data set for population analyses. This will have the effect of underestimating some measures of diversity and blunting the precision of population analyses to a small extent.

The number of alleles among 11 loci ranged from 5 to 17 and averaged 11.3 per locus (Table 4, Table S16 in Appendix S1). Allelic richness ranged from 3.06 to 8.52 among loci and averaged 5.34. The number of private alleles ranged from 0 to 16 and averaged 3.7 per sample. *H*_E_ ranged from 0.0132 to 0.460 and averaged 0.360. *F*_IS_ ranged from −0.052 (heterozygote excess) to 0.096 (heterozygote deficit) per sample, except for one population for which it was 0.217, and averaged 0.038.

**TABLE 4:** Summary statistics for 11 microsatellite loci in 14 samples from the Gulf of Alaska and Southeast Bering Sea

Location	*N*	*N*_A_	*N*_AR_	*N*_PA_	*H*_O_	*H*_E_	*F*_IS_
1	37.0	1.73	1.52	1	0.136	0.145	0.059
2	85.9	6.45	4.16	16	0.482	0.498	0.033
3	27.7	2.64	3.39	0	0.378	0.379	0.003
4	31.8	3.82	3.41	4	0.457	0.436	−0.050
5	30.0	4.73	3.96	2	0.402	0.455	0.096
6	24.2	3.91	3.67	0	0.399	0.398	0.001
7	20.4	4.09	3.82	3	0.478	0.483	0.010
9	16.5	4.27	4.01	4	0.287	0.364	0.217
10	25.9	2.73	2.33	2	0.284	0.289	0.017
11	31.3	3.00	2.75	4	0.464	0.464	−0.052
12	27.1	4.73	4.10	8	0.432	0.441	0.086
13	26.6	3.45	3.00	2	0.373	0.377	0.038
14	39.5	3.45	2.94	2	0.325	0.325	0.071
Mean	32.8	3.77	3.31	3.7	0.377	0.391	0.038
Pooled	423.9	49.00	11.21	48	0.387	0.605	0.361

Note

*N* = mean sample size over loci. *N*_A_ = mean number of alleles. *N*_AR_ = mean allelic richness with 28 alleles. *N*_PA_ = number of private alleles. *H*_O_ = observed heterozygosity. *H*_E_ = expected heterozygosity. *F*_IS_ = inbreeding coefficient. *N*_AR_ for the pooled sample was estimated with resamples of 824 alleles. Sample numbers as in Table 1.

*F*_ST_ between populations varied from 0.009 between locations Homer Spit (5) and Humpy Creek (6), both in Kachemak Bay, to 0.675 between distantly separated Nateen Bay (1) and Cordova (9) (Table 3, upper triangle). All pairwise comparisons were significantly greater than 0.0 (*p* < 0.001), except between populations 5 and 6 in Kachemak Bay (*p* = 0.054). Overall, *F*_ST_ = 0.239 (*p* < 0.0001) among populations. AMOVA indicated that 23.9% of the variability was due to allele frequency differences among populations, on average, and 76.1% was due to differences among individuals within populations (Table S16 in Appendix S1). Mantel's correlation between genetic divergence (*F*_ST_) and geographic distance (km) was *r* = 0.231 (*p* < 0.080) (Figure 3c) and between *F*_ST_ and log(km) was *r* =0 .401 (*p* = 0.005) (Figure 3d). Back assignments of individuals to populations of origin were largely accurate (Table 5). Four samples (1, 3, 4, and 11) showed perfect assignments, and five samples (2, 7, 9, 10, and 12) showed small numbers of misassignments to distant locations. The largest number of misassignments occurred between two pairs of neighboring locations 5–6 and 13–14.

**TABLE 5:** Log‐likelihood (*p* < 0.01) assignments of plants to populations based on allelic frequencies of 11 microsatellite loci in sugar kelp in the Gulf of Alaska

1	38	1	–	–	–	–	–	1	1	–	2	–	–
2	–	89	–	–	–	–	–	–	–	–	–	–	–
3	–	–	30	–	–	–	2	–	–	–	–	–	–
4	–	–	–	32	–	–	–	–	–	–	–	–	–
5	–		–	–	20	8	–	–	–	–	–	–	–
6	–	1	–	–	11	17	–	–	–	–	–	–	–
7	–	–	–	–	–	–	22	–	–	–	–	–	–
9	–	–	–	–	–	–	1	24	–	–	–	–	–
10	–	–	–	–	–	–	–	–	30	–	–	–	–
11	–	–	–	–	–	–	–	–	–	32	–	–	–
12	–	–	–	–	–	–	–	–	–	–	29	–	–
13	–	1	–	–	–	–	–	–	–	–	–	25	1
14	–	–	–	–	–	–	–	–	–	–	–	3	39
	1	2	3	4	5	6	7	9	10	11	12	13	14
Location number													

Note

Assignments are from populations on the *x*‐axis to populations on the *y*‐axis. Assignments back to original populations are in the diagonal. Location numbers as in Table 1.

**FIGURE 5:**
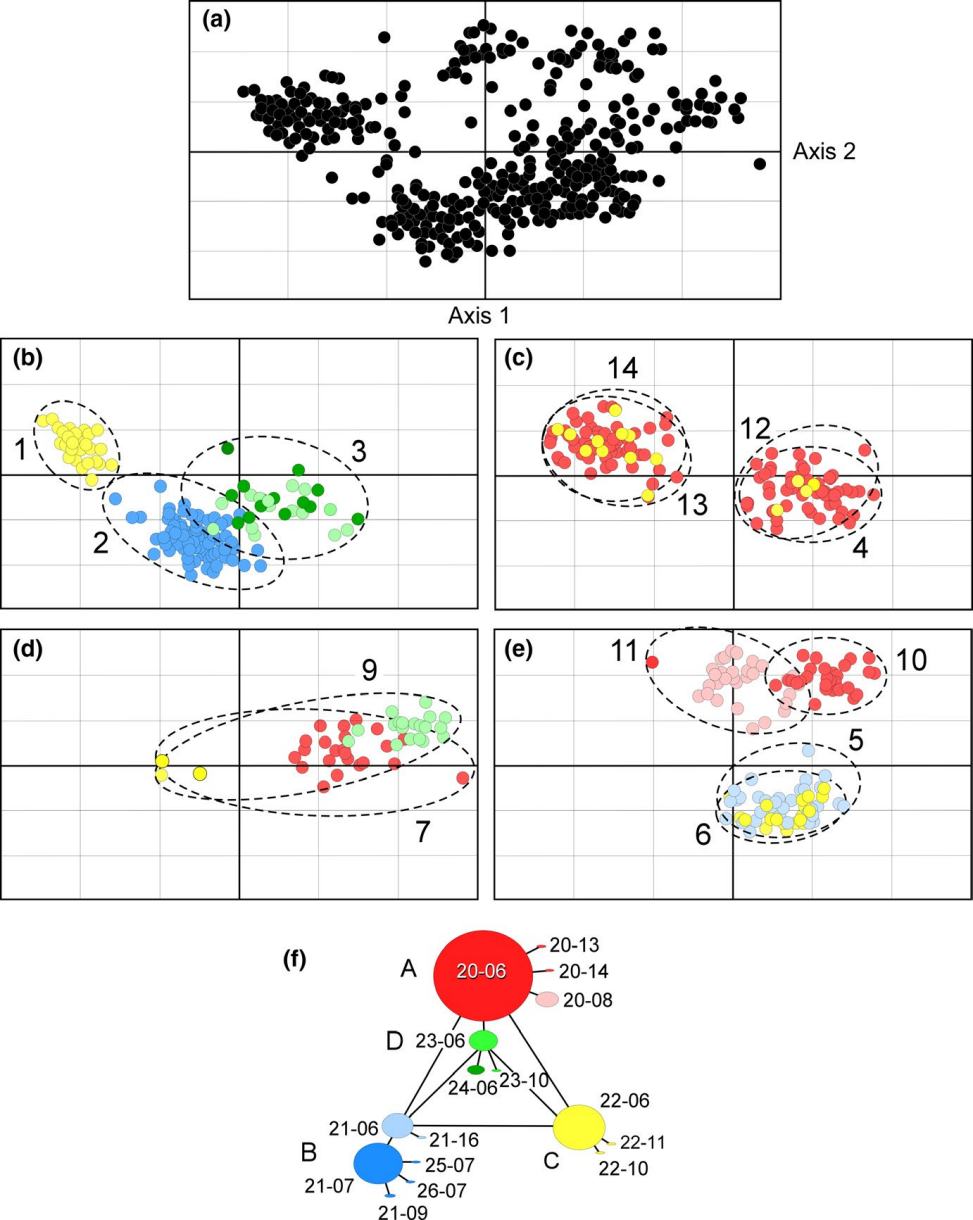
Principal coordinate analysis (PCoA) of microsatellite DNA (11 loci) allele frequencies sugar kelps, *Saccharina latissima*, in Alaska. (a) Total PCoA of samples from 13 sites in the Gulf of Alaska and southeastern Bering Sea. (b‐e) Individual locations to better illustrate genetic relationships among populations. (f) Haplotype network of concatenated *rbc*L and *COI* sequences from Figure 2f. Location numbers as in Table 1

A PCoA of microsatellite allele‐frequency variability among individuals resolved several population clusters. Separations defined by axes 1 and 2 accounted for only 25.4% of the variability in 13 samples (Figure 5a). Axis 3 accounted for only an additional 6.7% of the variability. In Figure 5b‐e, the PCoA results were separated by sample location to better visualize geography, and individuals were tagged with *rbcL*‐*COI* lineages in colors corresponding to those in Figure 2e. The geographic distributions of organellar lineages and the microsatellite population clusters differed among populations. Some of the microsatellite clusters consisted of a single *rbc*L‐*COI* lineage, but others included more than one lineage. Location 3 included 2 closely related haplotypes in lineage **D**, and widespread locations 4, 5, 6, 7, 9, and 14 contained haplotypes of lineage **C**. Locations 9 and 11 contained low frequencies of lineage **A**.

Kelps at locations 1 (lineage **C**), 2 (**B**), and 11 (**A**) were distinguishable from other locations by both microsatellites (*F*_ST_: 0.179–0.675) and *rbc*L‐*COI* DNA (Φ_ST_: 0.356–0.994). Kelps at locations 5 and 6 (both **B‐C** *)* in Kachemak Bay were indistinguishable from each other with both *rbc*L‐*COI* (Φ_ST_ = 0.0) and microsatellites (*F*_ST_ = 0.0). Locations 3 and 9 shared haplotypes in the same lineage (**D**) but were distinguishable by both *rbc*L‐*COI* (Φ_ST_ = 0.356) and microsatellites (*F*_ST_ = 0.404). Locations 10, 12, and 13 were fixed with the same *rbc*L*‐COI* haplotype (**A**) (Φ_ST_ = 0.0) but differed significantly from each other in microsatellite allelic frequencies (*F*_ST_: 0.326–0.470).

Haplotypes in lineage **C** were widespread among locations across the Gulf of Alaska and co‐occurred with lineages **A** at locations 4, 7, and 14, with **B** at locations 5 and 6, and with **D** at location 9. At these locations, kelps clustered together regardless of *rbc*L‐*COI* lineage. At location 9, a single kelp with *rbc*L‐*COI* **C** occurred among kelps carrying **D**‐lineage haplotypes, but did not show microsatellite convergence (Figure 5d).

**FIGURE 6:**
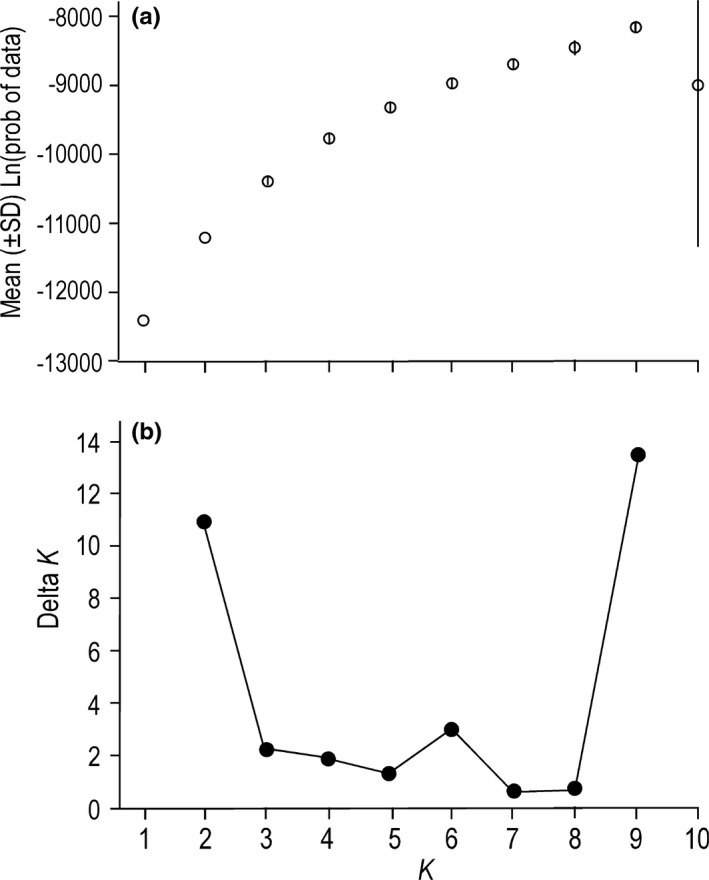
Estimates of the number of population cluster, *K*, at 11 microsatellite loci in sugar kelp, *Saccharina latissima*, at 13 locations in Alaska. (a) Distribution of mean values (10 replicates) of the likelihood of the fit of the model to the data. (b) Distribution of Δ*K* among 10 replicate runs over 10 test values of *K*

**FIGURE 7:**
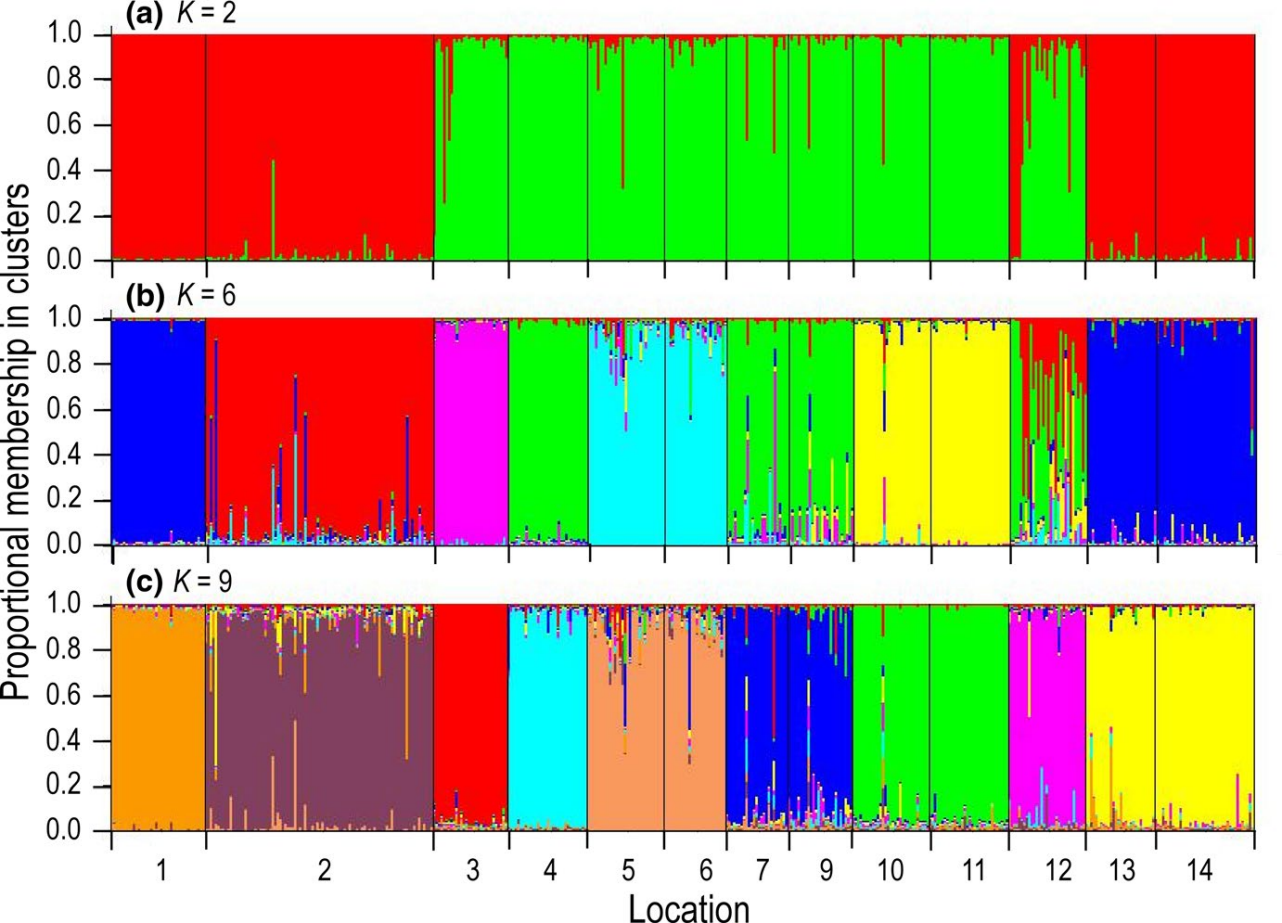
Linear STRUCTURE plots of admixture in individual sugar kelps, *Saccharina latissima*, at 13 locations in Alaska. (a) Estimated *K* = 2 number of population clusters. (b) *K* = 6. (c) *K* = 9. Location numbers correspond to number in Table 1

The results of the STRUCTURE analyses were ambiguous in that no clear number of genetic population clusters, *K*, were identified. The means of the ln(probability) among 10 replicate runs for test values of *K* did not plateau to indicate a likely value of *K* (Figure 6a). Peaks in the Δ*K* statistic appeared for *K* = 2, 6, and 9 (Figure 6b). Hence, the admixture plots with the largest likelihoods among the 10 replicates for each of the three values of *K* are presented in Figure 7. At *K = *2, two geographically separated groups of populations appeared with one group divided between western locations 1 and 2 and eastern locations 13 and 14 (Figure 7a). Location 12 contained kelps with ancestries of both groups. The *K* = 6 plot also showed a discontinuously distributed population cluster with members in the far western (1) and far eastern (13, 14) areas of the Gulf of Alaska (Figure 7b). Another group was discontinuously distributed among locations 4, 7, and 9, and was present to a small degree at location 12, which also showed heterogeneous ancestries in the *K* = 2 plot. Locations 5 and 6 were included in the same cluster. Locations 10 and 11, which were in the same general region, were also included in the same ancestral cluster. Locations 2 and 3 each consisted of a unique ancestral group. At *K* = 9, three pairs of locations, 5–6, 10–11, and 13–14, each showed the same ancestry. The rest of the population clusters included only a single location.

## 4 DISCUSSION

The cornerstone of phylogeographic inference is the analysis of geographic variability in organellar genes that do not undergo recombination during gamete formation and that are inherited uniparentally (Avise, 2000). In our study, we included both mitochondrial and plastid DNA markers, which fulfill these two requirements. However, the use of these markers alone limits the kinds of insights that can be made about population dynamics, such as the extent of hybridization between lineages. Hence, we also include 11 microsatellite loci in our study. Mitochondrial and chloroplast genes and microsatellite markers are inherited independently of one another, so these markers together provide a multifaceted view of phylogeographic history. Another strength of our study is the wide geographical coverage (2,800 km) and the analyses of larger sample sizes than are customary in studies of seaweeds, which have largely targeted taxonomic hypotheses (e.g., Lane et al., 2007). We follow with discussions of the molecular markers themselves and then with the pattern of population structure these markers depict. The results reveal a complex population history arising from repeated isolations in northern glacial refugia and postglacial expansions.

Contrary to the results for other seaweeds (Grant, 2016), the plastid gene *rbc*L in sugar kelp is more polymorphic than mitochondrial *COI* in sugar kelp and provides a greater amount of phylogeographic information. Notably, transitions and transversions at a single nucleotide site defined the four major lineages, **A**, **B**, **C**, and **D**. This site is located in the third “wobble” position of a codon encoding the nonpolar amino acids methionine and isoleucine: ATG produces methionine, and ATT, ATC, and ATA encode isoleucine. The latter three polymorphisms are silent and hence are unconstrained by natural selection, allowing for a higher level of polymorphism than polymorphisms leading to amino acid changes. Mutations at this site have occurred multiple times at various locations around the Gulf of Alaska and mark major geographic partitions. Why this particular nucleotide site is more polymorphic than other third‐position sites is uncertain.

One concern in the choice of microsatellite markers is the presence of a sufficient amount of diversity to detect population structure. 12 microsatellite loci were initially described by Paulino et al. (2016). Of these, 11 loci were used in our study and in studies by Neiva et al. (2018) and Næss (2019). 10 of these loci were used by Breton et al. (2018). Average heterozygosity in Alaskan populations (*H*_E_ = 0.391) was similar to the mean values for 2 populations in British Columbia (*H*_E_ = 0.349; Neiva et al., 2018) (Table S18 in Appendix S1). In the Northwest Atlantic, mean values of heterozygosity among populations were similar to those in the NE Pacific (*H*_E_ = 0.413, Neiva et al., 2018; *H*_E_ = 0.305, Breton et al., 2018). In the NE Atlantic along coastal Europe, estimates of heterozygosity were larger (*H*_E_ = 0.547, Paulino et al., 2016; *H*_E_ = 0.531, Neiva et al., 2018; *H*_E_ = 0.570, Næss, 2019). Even though the levels of microsatellite variability in the NE Pacific were lower than in other regional populations of sugar kelp, they were still large enough to resolve population structure.

Another concern with the use of microsatellite markers is the presence of null alleles. Values of *H*_E_ in our study exceeded *H*_O_ in 9 of the 13 samples, producing values of *F*_IS_ from −0.052 (heterozygote excess) to 0.096 (heterozygote deficit) per sample, except for one population for which it was 0.217, and averaged 0.038. These relatively small inbreeding coefficients and the general lack of departures from Hardy–Weinberg proportions indicate that null alleles did not consistently occur at high frequencies at any particular locus. Furthermore, a component of the *F*_IS_ values may have been due to inbreeding (Chybicki & Burczyk, 2009), as restricted spore dispersal in the subdivided kelp populations limits outbreeding. The high level of correct individual assignments back to originating populations also gives further confidence in the microsatellite data (Table 5).

### 4.1 Contemporary population structure

An understanding of the genetic background of kelps is important for the development of commercial cultivars (Goecke et al., 2020). The extent that populations are connected through gene flow is an important consideration for the management of a developing seaweed industry in Alaskan waters. While no signal of IBD was detected among populations, the sharing of low‐frequency *rbc*L‐*COI* haplotypes between some neighboring locations reflects connectivity between locations. For example, locations 3, 4, 5, 6, 7, 9, 11, and 14 had low‐frequency haplotypes differing from the local common lineage, most likely indicating migration from other populations.

Levels of microsatellite divergence between populations varied considerably, but showed only a weak correlation between genetic and geographical distance (Figure 3b,c). The lack of divergence between adjacent populations can readily be explained by gene flow between them, as for example between populations 5 and 6 in Kachemak Bay, which appear to consist of a single population in the bay. The association between genetic and geographical distance, however, weakens between populations separated by geographical distances greater than about 300 km, indicating that population history has played a greater role in sculpting population structure on larger geographic scales in the Gulf of Alaska than has contemporary levels of gene flow between populations.

The microsatellite allele‐frequency similarity between these populations, which are separated by several hundred kilometers in some cases (Figure 5), is unlikely due to ongoing gene flow, given the poor ability of kelp meiospores and gametes to disperse long distances (Anderson & North, 1966; Gaylord et al., ,^2002^, ^2004^, ^2006^). Populations of sugar kelp around the Gulf of Alaska appear to have not yet reached drift–migration equilibrium after the last glacial maximum and likely have perpetually been in disequilibria over the many glacial cycles of the Pleistocene. A close association between organellar DNA lineages (*COI*) and population groups, based on microsatellite allele‐frequency variability, also appeared on both sides of the North Atlantic (Figure 4 in Luttikhuizen et al., 2018; Figure 4 in Neiva et al., 2018), indicating the general importance of historical isolations among populations in producing contemporary patterns of diversity among populations.

### 4.2 Temporal patterns of population clustering

The STRUCTURE analysis indicated three possible values of *K*, 2, 6, and 9 (Figure 7a‐c). Meirmans (2015) suggests that different values of *K* may reflect various levels of population structure. We suggest the three cluster plots for sugar kelp microsatellite loci are remnant signatures of past population structures. Accordingly, the two large clusters depicted in the *K* = 2 plot (Figure 7a) may represent one of two historical scenarios. If sugar kelp abundances had been greatly reduced in the past, the two groups may represent an ancient geographical partition into two major population groups. If, on the other hand, population abundances have been more or less stable over time, the two genetic groups reflect the survival of only two genetic clusters among many. The two geographically disjunctive population clusters are reminiscent of the phylogeographic patterns of *COI* variability (Figure 2b). *COI* lineage **C** is scattered in patches across the Gulf of Alaska, loosely reflecting the disjunct microsatellite cluster split between the eastern Aleutians and Southeast Alaska 2,000 km away.

The admixture plots at larger values of *K* resolve populations into finer population units, possibly reflecting a progression through time. The STRUCTURE plot for *K* = 6 (Figure 7b) parallels the pattern of subdivision depicted with cpDNA in having a similar number of geographically disjunctive groups. The patterns are not identical because organellar cpDNA and nuclear microsatellite loci have evolved independently of one another. The influences of gene flow, local founder effects, and random drift on patterns of genetic diversity differ from one population to another and from one molecular marker to another.

Partitioning of the microsatellite genotypes into 9 clusters provides the best fit of the data to the STRUCTURE model (Figures 6 and 7c). The number of population clusters is similar to the number of *rbc*L‐*COI* lineages (Figure 2b). Further, the geographically disjunctive microsatellite population clusters are similar to the disjunct distributions of the *rbc*L‐*COI* lineages. Both the *K* = 9 population clusters and *rbc*L‐*COI* lineages likely represent contemporary levels of connectivity between populations.

Four pairs of more or less neighboring populations, 5–6, 7–9, 10–11, and 13–14, were each placed in the same population cluster in all three STRUCTURE plots (Figure 7a‐c). This likely indicates gene flow between the populations in each pair over time has prevented these populations from diverging from each other. Considering only microsatellite variability, the allele‐frequency similarity between the populations in each pair indicates genetic neighborhoods of 10–100s of kilometers in extent in some areas. However, the *rbc*L‐*COI* sequences show divergences between populations in pairs 7–9, 10–11, and 13–14 of one mutation (Figures 2e,f and 4c‐e). Populations 5 and 6 are exceptions to this pattern because their geographic proximity in the same bay may facilitate large amounts of gene flow between them. This contrast between patterns of divergence in microsatellite and in *rbc*L‐*COI* markers cannot entirely be due to patterns of gene flow, but may also reflect differences in mutation rates.

The organellar DNA and microsatellite data together in these population pairs show different rates of evolution in the marker classes. In some cases, the *rbc*L‐*COI* lineages were the same in the population pairs but in other cases they were different. Common origins of two populations would be expected to start the populations in the same clusters with the same *rbc*L‐*COI* haplotypes and microsatellite allele frequencies. Unexpectedly, the organellar genes have evolved more rapidly than the microsatellite loci in the population pairs 7–9, 10–11, and 13–14 (Figures 2f, 5, and 7c). The haplotype frequency shift in clusters 10–11 occurred in the *COI* sequences, but shifts between the pairs 1–9 and 13–14 occurred in the highly variable *rbc*L nucleotide site mentioned above. In other cases, microsatellite loci have evolved faster than organellar genes at locations 4, 7, 10, 12, and 13, which differ from one another at microsatellite loci but still bear the same *COI‐rbc*L haplotype in lineage **A** (Figures 2f, 5, and 7c). Also, kelps at locations 3 and 9 carry lineage **D** haplotypes, but have diverged in microsatellite frequencies. These results support the contention that mutation rates in organellar genes are not generally larger than in nuclear genes, or vice versa (Karl et al., 2012).

### 4.3 Hybridizations between organellar DNA lineages

Of evolutionary interest is whether kelps in the various DNA lineages are reproductively isolated from one another. The PCoA of microsatellite variability showed convergence of kelp genotypes in different organellar gene lineages at the same locations. For example, locations 4 and 14 included kelps in lineages **A** and **C** and the microsatellite genotypes of kelps in the two lineages converged in the PCoA ordination (Figure 5). Kelps at locations 5 and 6 also included the divergent lineages **C** and **B**, but again clustered together in the microsatellite PCoA indicating hybridizations between these two *rbc*L‐*COI* lineages. An exception occurred at Cordova (location 9), which included a **C**‐lineage haplotype among **D** lineage kelps that did not show convergence in the PCoA plot. This likely reflects a recent migration of **C** lineage kelps into this population. In general, we would expect to find strong heterozygote deficits (Wahlund's effect) at microsatellite loci at locations where *rbc*L‐*COI* lineages co‐occurred if the divergent lineages were reproductively isolated, but this was not the case (Table 4).

The individual admixture plots from the STRUCTURE analysis provide further insights into patterns of hybridizations between different population clusters (Figure 7a‐c). Populations 1, 3, 4, and 11 have few individuals with admixed ancestries from other population clusters. Other population clusters contain individuals with considerable admixture, including population clusters 2, 5–6, 7–9, and 13–14, with the greatest amount of admixture in population 12 at *K* = 6 (Figure 7b). Remarkably, the source of admixture at location 12, which is located in SE Alaska, came from the SE Bering Sea, several hundred km away.

### 4.4 Climate reconstructions and possible glacial refugia

Details of the paleoclimatic history of the NE Pacific support the contention that kelp, along with other intertidal and shallow subtidal organisms, survived in northern glacial refugia. Several studies of paleoclimate show that some northern shores remained ice‐free as the margins of the Cordilleran ice sheet repeatedly flowed onto coastal areas of the NE Pacific Ocean over the Pleistocene Epoch (2.6–0.012 Ma). Large expanses of glaciers around the Gulf of Alaska and along the coast of British Columbia were fed by precipitation from moist air masses moving across the North Pacific Ocean from the west (COHMAP, 1988; Kutzbach et al., 1993). Importantly, the margins of the Cordilleran ice sheet were irregular. Lobes of the continental glaciers reached the continental shelf largely through deep‐cut fjords in the coastal mountain range, leaving numerous stretches of coastline ice‐free (Carrara et al., 2007; Clague & James, 2002; Kaufman & Manley, 2004; Kaufman et al., 2011; Mann & Hamilton, 1995; Mann & Peteet, 1994). Many of these ice‐free coastal areas were associated with nearby terrestrial refugia (Carrara et al., 2007; Heaton & Grady, 2003; Reimchen & Byun, 2005).

Despite coastal glaciers, environmental reconstructions indicate that oceanic conditions remained conducive to the growth of kelps around the Gulf of Alaska and along the coast of British Columbia. Ocean temperatures in the Gulf of Alaska were relatively warm, dropping 5–6°C from present temperatures during glacial maxima (COHMAP‐Members, 1988;  Kutzbach et al., 1993). Sea surface temperature (SST) reconstructions indicate that the NE Pacific was not covered in perennial sea ice during glacial maxima (COHMAP‐Members, 1988), although icebergs were common (Keigwin & Gorbarenko, 1992). Surface salinities of coastal waters were lower, retarding convection and lessening the mixing of surface waters with nutrient‐rich deep waters (Gong et al., 2019; Worne et al., 2019; Zahn et al., 1991). Even so, depressed levels of nutrients were unlikely to interrupt completion of the sugar kelp life cycle.

Farther to the west, glacier‐free shorelines around a smaller Bering Sea may also have served as glacial refugia. Lower sea levels during glacial maxima exposed the Bering Land Bridge, which remained unglaciated with ice‐free southern shores. Nevertheless, seasonal sea ice covered the Bering Sea (Sancetta, 1983; Sancetta et al., 1984), leading to environmental conditions similar to those along the seasonally ice‐covered shores of the Arctic Beaufort Sea, where sugar kelp presently occur (Bringloe et al., 2017, 2020). The unique haplotypes at Port Moller (2) in the Southeastern Bering (Figure 2) support the concept of a southern Beringian shoreline refugium.

### 4.5 Genetic signatures of northern refugia

A robust test of the diversity gradient hypothesis with microsatellites or organellar DNA is not possible because of the absence of samples from unglaciated areas of coastal Washington, Oregon, and California. However, five *COI* sequences (FJ409200–F409204) from kelps located between 48.36° and 49.84°N all consisted of the most abundant haplotype in the Gulf of Alaska (MT040306). These samples originated from coastal areas that were potentially covered by the margins of the Cordilleran ice sheet, and hence do not provide a rigorous test of the diversity–gradient hypothesis. In the absence of samples from southern latitudes, we argue from substantial levels of organellar and nuclear diversity and from emerging insights into the disjunct distributions of tidewater glaciers around the Gulf of Alaska that sugar kelp populations survived in northern refugia during glacial maxima.

Several genetic signatures of sugar kelp populations in the Gulf of Alaska are consistent with northern ice‐age refugia. First, although both mtDNA and cpDNA genealogies across the Gulf of Alaska are shallow with the combined *rbc*L‐*COI* haplotypes separated by only 1–5 mutations, these markers detected considerable diversity among populations (Φ_ST_ = 0.834, *p* < 0.0001) that cannot be interpreted as a postglacial expansion from a southern refuge. Eight of 14 populations were fixed, or nearly fixed, with different *rbc*L‐*COI* haplotypes. This population heterogeneity is inconsistent with dispersals from a southern refuge, but reflects isolations in several northern refugia.

Second, the relative timings of glacial retreats along NE Pacific would have influenced patterns of genetic diversity among present‐day populations of sugar kelp. The growth and decline of coastal glaciers were out of phase between northern and southern shores. In Alaska, glaciers reached maximal extents twice between 23 and 17 ka, and most coastal areas were ice‐free by 16 ka. However, at the southern edge of the Cordilleran ice sheet, glaciers did not retreat from southwestern British Columbia and Puget Sound until 14–13 ka (Clague & James, 2002; Ryder et al., 1989; Thorson, 1980). If surviving populations had been limited to a southern refuge, postglacial invasions of northern shores would have been unimpeded by northern coastal glaciers and rapid colonizations would have led to genetically homogeneous northern populations (Hewitt, 1996). This is not supported by the strong genetic heterogeneity among populations in the Gulf of Alaska.

Third, rapid population growth is expected when new habitats are colonized, producing an excess of low‐frequency mutations over that expected for stable populations (Avise, 2000). However, the *rbc*L and *COI* haplotype frequency distributions failed to show these telltale departures from neutrality (Table 1). The absence of genetic imprints of geographic expansion and “recent” population growth is underscored by deep divergences between lineages dating to one million years or more (Figure 4).

Northern glacial refugia in the NE Pacific for intertidal, or shallow subtidal, invertebrates and fishes have been well‐established. The genetic signatures of three ecologically important mollusks (*Mytilus trossulus*, *Mytilus californianus*, and *Katharina tunicate*), two barnacles (*Balanus glandula* and *Semibalanus cariosus*), a sea star (*Patria miniata*), and an intertidal blenny (*Xiphister atropurpureus*) were consistent with stable populations that had not been displaced to southern refugia during glaciations (Marko et al., 2010). The northern clingfish (*Gobbiesox meandricus*) shows high diversities in northern populations, also indicating persistence during glacial maxima in northern refugia (Hickerson & Ross, 2001). Survival in northern marine refugia has also been postulated for Pacific cod (Bigg, 2014; Canino et al., 2010).

Genetic signatures of northern refugia have also been found for several species of algae. In addition to sugar kelp, split kelp (*Hedophyllum nigripes*) (Grant et al., 2020) and wing kelp (*Alaria* “*marginata*” complex) (Grant & Bringloe, 2020) have a mosaic population structure and high levels of genetic diversity in the Gulf of Alaska that reflect persistence in several northern refugia. Some species in the kelp genus *Agarum* also have geographical distributions that are consistent with persistence in northern glacial refugia (Boo et al., 2011). Finally, an study of Pacific dulse (*Palmaria mollis*) using random amplified polymorphic DNA revealed a phylogeographic break between populations between SE Alaska and south‐central and western Alaska that likely indicates secondary contact between populations isolated in northern glacial refugia (Lindstrom et al., 1997). In fact, a break in the geographic distributions of several algae at Southeast Alaska indicates contact between northern species that had to have survived glaciations in Alaskan waters and southern species from southern refugia (Lindstrom, 2009). The genetic signatures of many intertidal and shallow subtidal marine algae indicate that local northern ice‐age refugia were common.

## 5 CONCLUSION

The pattern of divergence among populations estimated with organellar DNA differed somewhat from the pattern estimated with microsatellite markers, a contrast that appears to be due to an interaction between gene flow and mutation in the different markers. Unquestionably, restricted dispersal between populations has led to a fragmented genetic population structure that failed to produce a signal of isolation by distance in the *rbc*L‐*COI* markers and only a weak signal in the microsatellite markers. Superimposed on innate patterns of gene flow are chance mutations in the molecular markers and population upheavals from numerous ice ages in the Pleistocene producing genetic imprints of local extinctions and colonizations.

Our argument that sugar kelp survived episodes of glaciation in northern refugia rests on three findings. First, while we were not able to make a robust test of the diversity gradient hypothesis because of the lack of data for populations in areas below the southern boundary of the last ice sheet, we found a considerable amount of genetic diversity in the Gulf of Alaska that was not predicted by the diversity–gradient hypothesis. Second, the source of genetic diversity in the Gulf of Alaska consists of differences between populations and not among individuals within populations. Individuals in several of the populations carried the same haplotype, implying high levels of inbreeding, a history of bottlenecks in population size, or a metapopulation dynamic that erodes genetic variability, all of which might have played a role in shaping population structure. A third finding was the absence of star‐shaped haplotype networks in individual populations and a lack of departures from neutrality that would indicate a range expansion from a southern refuge. Hence, we hypothesized that the mosaic pattern of genetic differences in the Gulf of Alaska originated from divergences during isolations in refugia. Haplotypes coalesced about one million years ago, indicating that sugar kelp populations have been confronted with environmental upheavals over several Croll‐Milankovitch cycles, which have both created and diminished genetic diversity.

## CONFLICT OF INTEREST

None declared.

## AUTHOR CONTRIBUTIONS

**William Stewart Grant:** Conceptualization (lead); data curation (lead); formal analysis (lead); funding acquisition (lead); methodology (supporting); resources (lead); writing‐original draft (lead); writing‐review & editing (lead). **Erica Chenoweth:** Conceptualization (supporting); data curation (supporting); methodology (lead); writing‐review & editing (supporting).

## ETHICAL APPROVAL

Samples were collected under Alaska Department of Fish and Game authorization.

## Supporting information

Appendix S1

## Data Availability

DNA sequences can be found in GenBank *COI* Accession Nos. MT040306–MT040319 and rbcL Accession Nos. MT040320–MT040327. Microsatellite DNA genotypes available from the North Pacific Research Board Project 1618 and from Figshare https://doi.org/10.6084/m9.figshare.11833086.
