# Supplementary material for: Phylogeography of sugar kelp: Northern ice‐age refugia in the Gulf of Alaska
Source: Ecol Evol. 2021 Mar 19;11(9):4670–87. doi: 10.1002/ece3.7368 (PMC8093666; doi:10.1002/ece3.7368)
Supplement: Supplementary file 1 — Appendix S1 [file ECE3-11-4670-s001.docx]

**APPENDIX 1**

**TABLE S1**  PCR thermal amplification profiles of 11 microsatellite loci in sugar kelp *Saccharina latissima*

| Locus Name | PCR thermal profile |
| --- | --- |
| *SLN314*; *SLN510* | 95 ^o^C/4 min; 33 cycles of (95 ^o^C/40 sec + 58 ^o^C/40 sec + 72 ^o^C/40 sec); 72 ^o^C 20 min |
| *SLN319*; *SLN320*; S*LN34* | 95 ^o^C/4 min; 33 cycles of (95 ^o^C/40 sec + 56 ^o^C/40 sec + 72 ^o^C/40 sec); 72 ^o^C 20 min |
| *SLN32*; *SLN36* | 95 ^o^C/4 min; 33 cycles of (95 ^o^C/40 sec + 57 ^o^C/40 sec + 72 ^o^C/40 sec); 72 ^o^C 20 min |
| *SLN511* | 95 ^o^C/4 min; 35 cycles of (95 ^o^C/40 sec + 54 ^o^C/40 sec + 72 ^o^C/40 sec); 72 ^o^C 20 min |
| *SLN54* | 95 ^o^C/4 min; 34 cycles of (95 ^o^C/40 sec + 54 ^o^C/40 sec + 72 ^o^C/40 sec); 72 ^o^C 20 min |
| *SLN58*; *SLN62* | 95 ^o^C/4 min; 32 cycles of (95 ^o^C/40 sec + 54 ^o^C/40 sec + 72 ^o^C/40 sec); 72 ^o^C 20 min |

### TABLE S2 Microsatellite primer sequences and PCR dyes used to amplify alleles in sugar kelp *Saccharina latissima*

| Oligo Name | Sequence (5' to 3') | 5' Dye |
| --- | --- | --- |
| SLN314-F | CTGTGTGTGTTGTCGTACATCG | NED |
| SLN314-R | GGATTTCTTATTTGAGGGAGGG |  |
| SLN319-F | CGAAGGAAGTGAATGACAACAA | 6FAM |
| SLN319-R | GGTAGTTACGGATTGCGACAAG |  |
| SLN320-F | TACGATGGTTTATGGGTTAGGG | PET |
| SLN320-R | AGCGAACAACGAAGCAACTAAT |  |
| SLN32-F | GAGAAAACATGCCCAGGTCTA | PET |
| SLN32-R | GTATCGCTGTACCCTCCTCCT |  |
| SLN34-F | ACGAAGTGCTAATAATGTGCCG | NED |
| SLN34-R | GAGATAGCCCGACCACTGC |  |
| SLN35-F | GCGTATGAACAAAATGACCGTA | VIC |
| SLN35-R | TGTGAGTTCCTTTCTTGTGAGC |  |
| SLN36-F | CGAGACTTTTGGGTAGATTTCG | VIC |
| SLN36-R | CGCCTGCCTCTTGTCTAAGTA |  |
| SLN510-F | CCGTCTATGGCGAGAAAGAGAT | 6FAM |
| SLN510-R | ATCTTACCTGGGCACTTGCTTT |  |
| SLN511-F | ATGTCCTGACCTGACCTACAGC | 6FAM |
| SLN511-R | AATTCTGTGAACATTCGGGAGT |  |
| SLN54-F | GTGGTTGCTGTTGTTGCTGT | VIC |
| SLN54-R | CGAATAAAGACAAATCGGCTG |  |
| SLN58-F | GCGAAGAAACGAGGGTTACAT | NED |
| SLN58-R | CTGGGTTTGTCGAGTGTTGAT |  |
| SLN62-F | ACAAAGCGTTCTCAACCGAT | PET |
| LN62-R | CGACACCCTACACAATACGAAA |  |

**TABLE S3** Microsatellite-locus pairs showing apparent linkage across all populations tested with Fisher’s method of summarizing test results for linkages between pairs of loci in each population.

| Locus pair | X^2^ | d.f. | *P* |
| --- | --- | --- | --- |
| SLN36–SLN511 | 45.75 | 24 | 0.005 |
| SLN320–SLN510 | 33.97 | 22 | 0.049 |

**TABLE S4** Microsatellite-locus pairs showing apparent linkage disequilibrium in individual populations tested with contingency-table chi-square. 11 microsatellite loci. 13 populations. Burn-in = 10000. Batches = 100. Iteration per batch = 5000.

| Location | Locus-pair | *P* | SE |
| --- | --- | --- | --- |
| 2 Nateen Bay | SLN34–SLN510 | 0.039 | 0.008 |
|  | SLN36–SLN511 | <0.00001 | 0.00001 |
|  | SLN54–SLN314 | 0.023 | 0.005 |
|  | SLN58–SLN319 | 0.041 | 0.004 |
| 3 Kuiuk Bay | SLN62–SLN511 | 0.013 | 0.002 |
| 4 Malina Bay | SLN62–SLN314 | 0.011 | 0.001 |
|  | SLN34–SLN62 | 0.042 | 0.002 |
| 5 Homer Spit | SLN320–SLN510 | 0.023 | 0.003 |
|  | SLN510–SLN511 | 0.013 | 0.003 |
|  | SLN36–SLN58 | 0.026 | 0.001 |
| 6 Humpy Creek | SLN314–SLN34 | 0.050 | 0.002 |
|  | SLN32–SLN34 | 0.041 | 0.002 |
|  | SLN34–SLN511 | 0.032 | 0.004 |
| 11 Auke Bay | SLN36–SLN58 | 0.034 | 0.001 |
| 12 Harris Island | SLN36–SLN510 | 0.016 | 0.005 |
| 14 Kaguk Bay | SLN319–SLN511 | 0.026 | 0.001 |
|  | SLN510–SLN511 | 0.017 | 0.003 |

**TABLE S5**  Haplotype frequencies of mitochondrial DNA cytochrome oxidase I*-*5’ (624 bp) haplotypes in 14 samples from the Gulf of Alaska and southeastern Bering Sea (samples 1–14). Haplotype numbers as in Fig. 1a. Sample numbers as in Table 1.

|  |  | Location | | | | | | | | | | | | | | | | | |  |
| --- | --- | --- | --- | --- | --- | --- | --- | --- | --- | --- | --- | --- | --- | --- | --- | --- | --- | --- | --- | --- |
| Haplotype | Lineage | 1 | 2 | | 3 | 4 | 5 | | 6 | 7 | 8 | 9 | 10 | | 11 | 12 | 13 | 14 | |  |
| MT040306 | A | 38 | | . | 31 | 32 | | 31 | 29 | 25 | 6 | 21 | 31 | 1 | | 26 | 32 | | 42 |  |
| MT040307 | B | . | | 87 | . | . | | . | . | . | . | . | . | . | | . | . | | . |  |
| MT040308 | A | . | | . | . | . | | . | . | . | . | . | . | 30 | | . | . | | . |  |
| MT040309 | B | . | | 3 | . | . | | . | . | . | . | . | . | . | | . | . | | . |  |
| MT040310 | A | 1 | | . | . | . | | . | . | . | . | . | . | . | | . | . | | . |  |
| MT040311 | A | . | | . | . | . | | . | 1 | . | . | . | . | . | | . | . | | . |  |
| MT040312 | A | . | | . | . | . | | . | 1 | . | . | . | . | . | | . | . | | . |  |
| MT040313 | A | . | | . | . | . | | . | . | 1 | . | . | . | . | | . | . | | . |  |
| MT040314 | A | . | | . | . | . | | . | . | 1 | . | . | . | . | | . | . | | . |  |
| MT040315 | A | . | | . | . | . | | . | . | . | . | 1 | . | . | | . | . | | . |  |
| MT040316 | A | . | | . | . | . | | . | . | . | . | 1 | . | . | | . | . | | . |  |
| Total |  | 39 | | 90 | 31 | 32 | | 31 | 31 | 27 | 6 | 23 | 31 | 31 | | 26 | 32 | | 42 |  |

**TABLE S6** Estimates of genetic parameters for mitochondrial DNA cytochrome oxidase I-5’ (624 bp) in samples of sugar kelp from the Gulf of Alaska–southeastern Bering Sea (1–14). Location number, sample size (*N*), number of polymorphic nucleotide sites (*N*_poly_), number of haplotypes (*N*_H_), expected number of haplotypes under neutrality (*N*_EH_), number of private haplotypes (*N*_PH_), haplotype diversity (*h*, standard deviation), nucleotide diversity (θ_π_, standard deviation) and Tajima’s *D* (*P*: probability of null hypothesis of neutrality). Sample numbers as in Table 1.

| Location | *N* | *N*_poly_ | *N*_H_ | *N*_EH_ | *N*_PH_ | *h* ±SD | θ_π_ (%) ±SD | *D* | *P* |
| --- | --- | --- | --- | --- | --- | --- | --- | --- | --- |
| 1 | 39 | 1 | 2 | 1.17 | 1 | 0.051 **±**0.048 | 0.011 **±**0.023 | -1.126 | 0.122 |
| 2 | 90 | 1 | 2 | 1.26 | 3 | 0.065 **±**0.035 | 0.011 **±**0.026 | -0.784 | 0.197 |
| 3 | 31 | 0 | 1 | 1 | 1 | 0.0 – | 0.0 – | – | – |
| 4 | 32 | 0 | 1 | 1 | 0 | 0.0 – | 0.0 – | – | – |
| 5 | 31 | 0 | 1 | 1 | 0 | 0.0 – | 0.0 – | – | – |
| 6 | 31 | 2 | 3 | 1.42 | 2 | 0.127 **±**0.080 | 0.022 **±**0.037 | -1.506 | 0.037 |
| 7 | 27 | 2 | 3 | 1.47 | 2 | 0.145 **±**0.090 | 0.025 **±**0.041 | -1.512 | 0.044 |
| 8 | 6 | 0 | 1 | 1 | 0 | 0.0 – | 0.0 – | – | – |
| 9 | 23 | 2 | 3 | 1.53 | 1 | 0.170 **±**0.103 | 0.029 **±**0.045 | -1.515 | 0.042 |
| 10 | 31 | 0 | 1 | 1 | 0 | 0.0 – | 0.0 – | – | – |
| 11 | 31 | 1 | 2 | 1.20 | 1 | 0.065 **±**0.059 | 0.011 **±**0.026 | -1.145 | 0.131 |
| 12 | 26 | 0 | 1 | 1 | 0 | 0.0 – | 0.0 – | – | – |
| 13 | 32 | 0 | 1 | 1 | 0 | 0.0 – | 0.0 – | – | – |
| 14 | 42 | 0 | 1 | 1 | 0 | 0.0 – | 0.0 – | – | – |
| Mean | 33.7 | 0.64 | 1.64 | 1.15 | 0.79 | 0.040 – | 0.007 – | – | – |
| Pooled | 472 | 10 | 11 | 4.39 | – | 0.429 **±**0.024 | 0.079 **±**0.076 | -1.452 | 0.041 |

**TABLE S7** Sequence divergences (Φ_ST_; Tamura & Nei 1993) based on mitochondrial DNA cytochrome oxidase I (624 bp) between populations in the Gulf of Alaska and southeastern Bering Sea (samples 1–14). Overall, Φ_ST_ = 0.909 (*P* < 0.00005) among populations. Bold indicates genetic distances that are significantly larger than 0.0 (*P* < 0.00005). Significances determined with 50 000 permutations. Population numbers as in Table 1.

| 2 | 0.943 |  |  |  |  |  |  |  |  |  |  |  |  |
| --- | --- | --- | --- | --- | --- | --- | --- | --- | --- | --- | --- | --- | --- |
| 3 | 0.0 | **0.954** |  |  |  |  |  |  |  |  |  |  |  |
| 4 | 0.0 | **0.954** | 0.0 |  |  |  |  |  |  |  |  |  |  |
| 5 | 0.0 | **0.954** | 0.0 | 0.0 |  |  |  |  |  |  |  |  |  |
| 6 | 0.003 | **0.925** | 0.0 | 0.001 | 0.0 |  |  |  |  |  |  |  |  |
| 7 | 0.006 | **0.923** | 0.005 | 0.006 | 0.005 | 0.0 |  |  |  |  |  |  |  |
| 8 | 0.0 | **0.942** | 0.0 | 0.0 | 0.0 | 0.0 | 0.0 |  |  |  |  |  |  |
| 9 | 0.011 | **0.921** | 0.013 | 0.015 | 0.013 | 0.002 | 0.001 | 0.0 |  |  |  |  |  |
| 10 | 0.0 | **0.954** | 0.0 | 0.0 | 0.0 | 0.0 | 0.005 | 0.0 | 0.013 |  |  |  |  |
| 11 | **0.943** | **0.968** | **0.967** | **0.967** | **0.967** | **0.906** | **0.901** | **0.944** | **0.894** | **0.967** |  |  |  |
| 12 | 0.0 | **0.952** | 0.0 | 0.0 | 0.0 | 0.006 | 0.0 | 0.0 | 0.005 | 0.0 | **0.964** |  |  |
| 13 | 0.0 | **0.954** | 0.0 | 0.0 | 0.0 | 0.001 | 0.006 | 0.0 | 0.015 | 0.0 | **0.967** | 0.0 |  |
| 14 | 0.002 | **0.957** | 0.0 | 0.0 | 0.0 | 0.010 | 0.017 | 0.0 | 0.028 | 0.0 | **0.972** | 0.0 | 0.0 |
|  | 1 | 2 | 3 | 4 | 5 | 6 | 7 | 8 | 9 | 10 | 11 | 12 | 13 |
| Location | | | | | | | | | | | | | |

**TABLE S8**  Analysis of molecular variance (AMOVA) of mitochondrial DNA 5’-*cytochrome oxidase* (624 bp) sequence variability among populations in the Gulf of Alaska and southeastern Bering Sea.

| Partition | d.f. | Variance component | % of variance | *P* |
| --- | --- | --- | --- | --- |
| Among populations | 13 | 0.234 | 90.9 | <0.00001 |
| Within populations | 458 | 0.023 | 9.1 |  |
| Total | 471 | 0.257 | 100.0 |  |

**TABLE S9** Haplotype frequencies of ribulose-1,5-bisphosphate carboxylase/oxygenase large subunit-3’ (*rbc*L) (735 base-pair fragment) haplotypes in samples from the Gulf of Alaska and southeastern Bering Sea. Haplotype numbers as in Fig. 1b. Sample numbers as in Table 1.

|  |  | Location | | | | | | | | | | | | | | | | |
| --- | --- | --- | --- | --- | --- | --- | --- | --- | --- | --- | --- | --- | --- | --- | --- | --- | --- | --- |
| Haplotype | Lineage | 1 | 2 | 3 | 4 | 5 | | 6 | | 7 | 8 | 9 | 10 | 11 | 12 | 13 | 14 |  |
| MT040320 | A | . | . | . | 28 | . | . | | 27 | | . | . | 29 | 27 | 23 | 31 | 32 |  |
| MT040321 | B | . | 78 | . | . | 20 | 17 | | . | | . | . | . | . | . | . | . |  |
| MT040322 | C | 37 | . | . | 4 | 10 | 15 | | 1 | | 6 | 1 | . | . | . | . | 10 |  |
| MT040323 | D | . | . | 16 | . | . | . | | . | | . | 21 | . | . | . | . | . |  |
| MT040324 | D | . | . | 14 | . | . | . | | . | | . | . | . | . | . | . | . |  |
| MT040325 | B | . | 2 | . | . | . | . | | . | | . | . | . | . | . | . | . |  |
| MT040326 | B | . | 1 | . | . | . | . | | . | | . | . | . | . | . | . | . |  |
| Total |  | 37 | 81 | 30 | 32 | 30 | 32 | | 28 | | 6 | 22 | 29 | 27 | 23 | 31 | 42 |  |

**TABLE S10** Estimates of genetic parameters for ribulose-1,5-bisphosphate carboxylase/oxygenase large subunit-3’ (*rbc*L) (735 base pairs) in 14 samples from the Gulf of Alaska and southeastern Bering Sea (samples 1–14). Location number, sample size (*N*), number of polymorphic nucleotide sites (*N*_poly_), number of haplotypes (*N*_H_), expected number of haplotypes under neutrality (*N*_EH_), number of private haplotypes (*N*_PH_), haplotype diversity (*h* **±** standard deviation), nucleotide diversity (θ_π_ **±** standard deviation) and Tajima’s *D* (*P*: probability of null hypothesis of neutrality). Location numbers as in Table 1.

| Location | *N* | *N*_poly_ | *N*_H_ | *N*_EH_ | *N*_PH_ | *h* ±SD | θ_π_ (%) ±SD | *D* | *P* |
| --- | --- | --- | --- | --- | --- | --- | --- | --- | --- |
| Gulf of Alaska | | | | | | | | | |
| 1 | 37 | 0 | 1 | 1.00 | 0 | 0.0 – | 0.0 – | – | – |
| 2 | 81 | 2 | 3 | 1.29 | 2 | 0.073 ±0.040 | 0.010 ±0.022 | -1.310 | 0.052 |
| 3 | 30 | 1 | 2 | 3.48 | 1 | 0.515 ±0.027 | 0.070 ±0.067 | 1.621 | 0.974 |
| 4 | 32 | 1 | 2 | 1.81 | 0 | 0.226 ±0.088 | 0.031 ±0.041 | -0.138 | 0.319 |
| 5 | 30 | 1 | 2 | 3.06 | 0 | 0.460 ±0.061 | 0.063 ±0.063 | 1.280 | 0.913 |
| 6 | 32 | 1 | 2 | 3.53 | 0 | 0.514 ±0.025 | 0.070 ±0.067 | 1.634 | 0.974 |
| 7 | 28 | 1 | 2 | 1.22 | 0 | 0.071 ±0.065 | 0.001 ±0.022 | -1.151 | 0.134 |
| 8 | 6 | 0 | 1 | 1.00 | 0 | 0.0 – | 0.0 – | – | – |
| 9 | 22 | 1 | 2 | 1.26 | 0 | 0.091 **±**0.081 | 0.012 ±0.026 | -1.162 | 0.151 |
| 10 | 29 | 0 | 1 | 1.00 | 0 | 0.0 – | 0.0 – | – | – |
| 11 | 27 | 0 | 1 | 1.00 | 0 | 0.0 – | 0.0 – | – | – |
| 12 | 23 | 0 | 1 | 1.00 | 0 | 0.0 – | 0.0 – | – | – |
| 13 | 31 | 0 | 1 | 1.00 | 0 | 0.0 – | 0.0 – | – | – |
| 14 | 42 | 1 | 2 | 2.65 | 0 | 0.372 **±**0.070 | 0.051 ±0.055 | 0.844 | 0.860 |
| Mean | 32.1 | 0.6 | 1.6 | 1.74 | 0.2 | 0.165 – | 0.022 – | – | – |
| Pooled | 450 | 4 | 7 | 10.50 | 3 | 0.702 **±**0.013 | 0.104 ±0.085 | 0.453 | 0.716 |

**TABLE S11** Genetic distances (Φ_ST_) between samples from the Gulf of Alaska, southeastern Bering Sea, based on sequence variability in ribulose-1,5-bisphosphate carboxylase/oxygenase large subunit (*rbc*L) (735 bp) with Tamura (1992) model of mutation. Overall, Φ_ST_ = 0.788 (*P* < 0.00005) among 14 populations in the Gulf of Alaska. Italics 0.05 > *P* > 0.01; Bold *P* < 0.01. Significances determined with 50 000 permutations. Sample numbers as in Table 1.

| 2 | 0.952 |  |  |  |  |  |  |  |  |  |  |  |  |
| --- | --- | --- | --- | --- | --- | --- | --- | --- | --- | --- | --- | --- | --- |
| 3 | **0.841** | **0.864** |  |  |  |  |  |  |  |  |  |  |  |
| 4 | **0.880** | **0.885** | **0.750** |  |  |  |  |  |  |  |  |  |  |
| 5 | **0.681** | **0.380** | **0.668** | **0.645** |  |  |  |  |  |  |  |  |  |
| 6 | **0.536** | **0.524** | **0.649** | **0.607** | 0.005 |  |  |  |  |  |  |  |  |
| 7 | **0.968** | **0.930** | **0.796** | 0.018 | **0.726** | **0.69**1 |  |  |  |  |  |  |  |
| 8 | **0.000** | **0.935** | **0.728** | **0.793** | *0.508* | *0.350* | **0.939** |  |  |  |  |  |  |
| 9 | **0.965** | **0.925** | **0.380** | **0.830** | **0.700** | **0.662** | **0.920** | **0.925** |  |  |  |  |  |
| 10 | **1.000** | **0.948** | **0.822** | 0.090 | **0.767** | **0.733** | 0.001 | **1.000** | **0.991** |  |  |  |  |
| 11 | **1.000** | **0.947** | **0.817** | 0.085 | **0.760** | **0.726** | 0.0 | **1.000** | **0.959** | 0.0 |  |  |  |
| 12 | **1.000** | **0.945** | **0.805** | 0.075 | **0.746** | **0.711** | 0.0 | **1.000** | **0.956** | 0.0 | 0.0 |  |  |
| 13 | **1.000** | **0.949** | **0.827** | 0.095 | **0.773** | **0.740** | 0.004 | **1.000** | **0.962** | 0.0 | 0.0 | 0.0 |  |
| 14 | **0.744** | **0.824** | **0.704** | 0.553 | **0.553** | **0.507** | *0.117* | **0.624** | **0.732** | *0.185* | *0.180* | *0.167* | *0.191* |
|  | 1 | 2 | 3 | 4 | 5 | 6 | 7 | 8 | 9 | 10 | 11 | 12 | 13 |

Location

**TABLE S12** Analysis of molecular variance (AMOVA) of ribulose-1,5-bisphosphate carboxylase/oxygenase large subunit (*rbc*L) (735 base pairs) sequence variability among samples from the Gulf of Alaska and southeastern Bering Sea with the Tamura (1992) model of mutation.

| Partition | d.f. | Variance component | % of variance | *P* |
| --- | --- | --- | --- | --- |
| Among populations | 13 | 0.325 | 78.8 | <0.00001 |
| Within populations | 436 | 0.087 | 21.2 |  |
| Total | 449 | 0.412 | 100.0 |  |

**TABLE S13** Analysis of molecular variance (AMOVA) of concatenated fragments of mitochondrial DNA cytochrome oxidase I-5’ (*COI*) and ribulose-1,5-bisphosphate carboxylase/oxygenase large subunit-3’ (*rbc*L) (1359 bp) among 14 samples from the Gulf of Alaska and southeastern Bering Sea with the Tamura (1992) model of mutation. Significance determined with 50 000 permutations.

| Partition | d.f. | Variance component | % of variance | *P* |
| --- | --- | --- | --- | --- |
| Among populations | 13 | 0.542 | 83.4 | <0.00001 |
| Within populations | 432 | 0.108 | 16.6 |  |
| Total | 445 | 0.650 | 100.0 |  |

**TABLE S14** Null-allele frequencies estimated by GENPOP. Dashes indicate loci with insufficient information to estimate null allele frequency. These loci had a frequency of 1.0 in a particular sample.

|  |  |  |  |  |  | Location | | |  |  |  |  |  |
| --- | --- | --- | --- | --- | --- | --- | --- | --- | --- | --- | --- | --- | --- |
| Locus | 1 | 2 | 3 | 4 | 5 | 6 | 7 | 9 | 10 | 11 | 12 | 13 | 14 |
| SLN32 | – | 0.063 | – | 0 | 0.139 | 0 | 0 | – | – | – | – | 0 | 0 |
| SLN34 | 0 | 0.061 | 0 | 0.021 | 0.114 | 0 | 0.010 | 0.197 | – | 0.107 | 0 | 0 | 0 |
| SLN36 | 0 | 0.027 | 0.041 | 0.009 | 0 | 0 | 0 | 0 | 0.075 | 0 | 0.020 | 0.133 | 0 |
| SLN54 | 0 | 0.031 | 0.027 | – | – | – | 0 | 0 | 0 | 0 | 0.062 | 0 | 0 |
| SLN58 | 0.101 | 0 | – | – | 0 | 0 | – | 0 | 0.037 | 0 | 0.083 | 0 | – |
| SLN62 | – | – | 0.039 | 0 | 0.002 | 0.101 | 0.071 | 0 | 0 | 0 | 0.047 | 0 | 0 |
| SLN314 | 0 | 0 | 0.050 | 0.018 | 0.022 | 0 | 0.034 | 0 | 0 | 0.028 | 0 | 0.082 | 0.073 |
| SLN319 | – | 0 | 0 | 0.018 | 0 | 0 | 0.072 | 0.190 | – | 0.019 | 0.115 | 0 | 0.040 |
| SLN320 | 0 | 0.018 | 0.039 | 0 | 0.039 | 0 | 0.028 | – | 0 | 0 | 0 | 0 | 0.085 |
| SLN510 | – | 0 | 0 | 0.020 | 0 | 0 | 0 | 0 | 0 | 0 | 0.080 | 0.007 | 0 |
| SLN511 | – | 0.013 | 0.013 | 0.060 | 0 | 0.021 | 0 | 0.142 | 0 | 0 | 0.116 | 0.000 | 0.031 |

**TABLE S15** Null-allele frequencies estimates by ML-NULL (Kalinowski & Taper 2016)

|  |  |  |  |  |  | Location | | |  |  |  |  |  |
| --- | --- | --- | --- | --- | --- | --- | --- | --- | --- | --- | --- | --- | --- |
| Locus | 1 | 2 | 3 | 4 | 5 | 6 | 7 | 9 | 10 | 11 | 12 | 13 | 14 |
| SLN32 | 0.198 | 0.155 | 0 | 0 | 0.119 | 0 | 0 | 0.615 | 0.18 | 0 | 0.331 | 0 | 0 |
| SLN34 | 0 | 0.081 | 0 | 0.024 | 0.114 | 0 | 0.013 | 0.441 | 0.417 | 0.183 | 0 | 0 | 0 |
| SLN36 | 0 | 0.033 | 0.041 | 0 | 0 | 0 | 0 | 0 | 0.12 | 0 | 0.02 | 0.198 | 0 |
| SLN54 | 0 | 0.028 | 0.027 | 0 | 0 | 0.177 | 0 | 0 | 0 | 0 | 0.084 | 0 | 0 |
| SLN58 | 0.101 | 0 | 0 | 0 | 0 | 0 | 0.306 | 0 | 0.059 | 0 | 0.202 | 0 | 0.158 |
| SLN62 | 0 | 0.254 | 0.002 | 0 | 0.002 | 0.101 | 0.090 | 0 | 0 | 0 | 0 | 0 | 0 |
| SLN314 | 0 | 0 | 0.050 | 0 | 0.022 | 0 | 0.040 | 0 | 0 | 0.028 | 0 | 0.115 | 0.073 |
| SLN319 | 0.140 | 0 | 0 | 0.018 | 0 | 0 | 0.095 | 0.353 | 0.433 | 0.030 | 0.027 | 0 | 0.069 |
| SLN320 | 0 | 0.011 | 0.039 | 0 | 0.039 | 0 | 0.038 | 0.578 | 0 | 0 | 0 | 0 | 0.085 |
| SLN510 | 0.106 | 0 | 0 | 0.004 | 0 | 0 | 0 | 0 | 0 | 0 | 0.100 | 0.008 | 0 |
| SLN511 | 0.279 | 0.014 | 0.013 | 0.060 | 0 | 0.023 | 0 | 0.202 | 0 | 0 | 0.151 | 0 | 0.039 |

**TABLE S16** Summary statistics of variability at 11 microsatellite loci pooled over 13 samples (no data for sample 8) from the Gulf of Alaska and southeastern Bering Sea. Number of plants (*N*), Number of alleles (*N*_A_), allelic richness (*N*_AR_) based on resampling 28 alleles, observed heterozygosity (*H*_O_), expected heterozygosity assuming Hardy-Weinberg proportions (*H*_E_), population inbreeding coefficient (*F*_IS_), differentiation among populations (*F*_ST_).

| Locus | *N* | *N*_A_ | *N*_AR_ | *H*_O_ | *H*_E_ | *F*_IS_ | *F*_ST_ |
| --- | --- | --- | --- | --- | --- | --- | --- |
| SLN32 | 435 | 6 | 3.06 | 0.126 | 0.222 | 0.069 | 0.413 |
| SLN34 | 413 | 12 | 4.45 | 0.378 | 0.615 | 0.105 | 0.335 |
| SLN36 | 440 | 17 | 8.52 | 0.602 | 0.769 | 0.034 | 0.205 |
| SLN54 | 412 | 14 | 5.20 | 0.296 | 0.544 | 0.005 | 0.477 |
| SLN58 | 429 | 5 | 3.20 | 0.184 | 0.378 | 0.095 | 0.487 |
| SLN62 | 429 | 6 | 3.56 | 0.296 | 0.442 | -0.086 | 0.408 |
| SLN314 | 432 | 10 | 5.48 | 0.495 | 0.756 | 0.055 | 0.328 |
| SLN319 | 433 | 15 | 5.26 | 0.419 | 0.711 | 0.035 | 0.469 |
| SLN320 | 420 | 9 | 5.15 | 0.379 | 0.726 | 0.012 | 0.440 |
| SLN510 | 438 | 15 | 6.50 | 0.484 | 0.663 | 0.009 | 0.284 |
| SLN511 | 414 | 15 | 8.39 | 0.589 | 0.831 | 0.051 | 0.271 |
| Mean | 426.8 | 11.3 | 5.34 | 0.376 | 0.605 | 0.035 | 0.372 |

**TABLE S17** Analysis of molecular variance (AMOVA) of *F*_ST_ based on microsatellite allele-frequency variability among 13 samples from the Gulf of Alaska and southeastern Bering Sea. Overall, *F*_ST_ = 0.239 (*P* < 0.0001)

| Partition | d.f. | Variance component | % of variance | *P* |
| --- | --- | --- | --- | --- |
| Among populations | 12 | 0.160 | 23.9 | <0.00001 |
| Among plants within populations | 907 | 0.507 | 76.1 |  |
| Total | 919 | 0.667 | 100.0 |  |

**TABLE S18** Comparison of microsatellite average expected heterozygosity among population genetic studies of sugar kelp, *Saccharina latissima*. Geographic ranges of sampling in the various studies were measure in Google Earth as approximate shoreline distances, or were taken from the original study.

| Region | No.  locations | Sample  range (km) | Range *H*_E_ | Mean *H*_E_ | Reference |
| --- | --- | --- | --- | --- | --- |
| NE Pacific | 14 | 2800 | 0.145–0.498 | 0.391 | Present study |
| NE Pacific | 2 | 700 | 0.324–0.375 | 0.349 | Neiva et al., 2018 |
| NW Atlantic | 15 | 4450 | 0.140–0.464 | 0.413 | Neiva et al., 2018 |
| NW Atlantic | 5 | 230 | 0.273–0.340 | 0.305 | Breton et al., 2018 |
| Arctic & NE Atlantic | 3 | 5100 | 0.478–0.615 | 0.547 | Paulino et al., 2016 |
| NE Atlantic | 7 | 5360 | 0.412–0.632 | 0.531 | Neiva et al., 2018 |
| NE Atlantic | 14 | 1360 | 0.445–0.635 | 0.570 | Næss, 2019 |
| NE Atlantic | 6 | 5100 | 0.204–0.243^a^ | 0.236^b^ | Guzinski et al., 2016 |

^a^Estimated from 32 EST-derived microsatellite loci embedded within coding genes

^b^Average among three southern populations
